# Painful gynecologic and obstetric complications of female genital mutilation/cutting: A systematic review and meta-analysis

**DOI:** 10.1371/journal.pmed.1003088

**Published:** 2020-03-31

**Authors:** Jacob Michael Lurie, Alessandra Weidman, Samantha Huynh, Diana Delgado, Imaani Easthausen, Gunisha Kaur

**Affiliations:** 1 Icahn School of Medicine at Mount Sinai, New York, New York, United States of America; 2 Department of Anesthesiology, Weill Cornell Medicine, New York, New York, United States of America; 3 Information, Education, and Clinical Services, Weill Cornell Medicine, New York, New York, United States of America; 4 Department of Healthcare Policy and Research, Weill Cornell Medicine, New York, New York, United States of America; Columbia University Mailman School of Public Health, UNITED STATES

## Abstract

**Background:**

The health complications experienced by women having undergone female genital mutilation/cutting (FGM/C) are a source of growing concern to healthcare workers globally as forced displacement and migration from countries with high rates of this practice increases. In this systematic review and meta-analysis, we investigate the association between FGM/C and painful gynecologic and obstetric complications in women affected by the practice.

**Methods and findings:**

We performed a comprehensive literature search from inception to December 19, 2019 of Ovid MEDLINE, Ovid EMBASE, The Cochrane Library (Wiley), and POPLINE (prior to its retirement) for studies mentioning FGM/C. Two reviewers independently screened studies reporting prevalences of painful gynecologic and obstetric sequelae resulting from FGM/C. Random effects models were used to estimate pooled odds ratios (ORs) for outcomes obtained from cross-sectional, cohort, and case–control designs. Subgroup analysis was performed to assess and control for effect differences introduced by study design. Validated appraisal tools were utilized to assess quality and risk of bias. Our study was registered with PROSPERO. Two reviewers independently screened 6,666 abstracts. Of 559 full-text studies assessed for eligibility, 116 met eligibility criteria, which included studies describing the incidence or prevalence of painful sequelae associated with FGM/C. Pooled analyses after adjustment for study design found that FGM/C was associated with dyspareunia (6,283 FGM/C and 3,382 non-FGM/C participants; pooled OR: 2.47; 95% confidence interval [CI]: 1.45–4.21; *I*^2^: 79%; *p*-value < 0.01), perineal tears (4,898 FGM/C and 4,229 non-FGM/C participants; pooled OR: 2.63; 95% CI: 1.35–5.11; *I*^2^: 67%; *p*-value = 0.01), dysuria (3,686 FGM/C and 3,482 non-FGM/C participants; pooled OR: 1.43; 95% CI: 1.17–1.75; *I*^2^: 0%; *p*-value = 0.01), episiotomy (29,341 FGM/C and 39,260 non-FGM/C participants; pooled OR: 1.89; 95% CI: 1.26–2.82; *I*^2^: 96%; *p*-value < 0.01), and prolonged labor (7,516 FGM/C and 8,060 non-FGM/C participants; pooled OR: 2.04; 95% CI: 1.27–3.28; *I*^2^: 90%; *p*-value < 0.01). There was insufficient evidence to conclude that there was an association between FGM/C and dysmenorrhea (7,349 FGM/C and 4,411 non-FGM/C participants; pooled OR: 1.66; 95% CI: 0.97–2.84; *I*^2^: 86%; *p*-value = 0.06), urinary tract infection (4,493 FGM/C and 3,776 non-FGM/C participants; pooled OR: 2.11; 95% CI: 0.80–5.54; *I*^2^: 90%; *p*-value = 0.10), instrumental delivery (5,176 FGM/C and 31,923 non-FGM/C participants; pooled OR: 1.18; 95% CI: 0.78–1.79; *I*^2^: 63%; *p*-value = 0.40), or cesarean delivery (34,693 FGM/C and 46,013 non-FGM/C participants; pooled OR: 1.51; 95% CI: 0.99–2.30; *I*^2^: 96%; *p*-value = 0.05). Studies generally met quality assurance criteria. Limitations of this study include the largely suboptimal quality of studies.

**Conclusions:**

In this study, we observed that specific painful outcomes are significantly more common in participants with FGM/C. Women who underwent FGM/C were around twice as likely as non-FGM/C women to experience dyspareunia, perineal tears, prolonged labor, and episiotomy. These data indicate that providers must familiarize themselves with the unique health consequences of FGM/C, including accurate diagnosis, pain management, and obstetric planning.

**Review protocol registration:**

The review protocol registration in PROSPERO is CRD42018115848.

## Abstract 2

In a systematic review and meta-analysis, Jacob Lurie and colleagues evaluate painful gynecologic and obstetric complications of female genital mutilation/cutting.

## Author summary

**Why was this study done?:**

- The health complications experienced by women having undergone female genital mutilation/cutting (FGM/C) are relevant to practitioners globally, who are increasingly likely to encounter patients with FGM/C because of amplified transnational migration.
- This study was done to analyze available data regarding pain outcomes and obstetric complications in the setting of FGM/C. Previous research has documented associations between FGM/C and outcomes that are considered painful, but such systematic reviews are scarce and, to our knowledge, have not been attempted in several years. Additionally, studies examining associations between FGM/C and painful outcomes may arrive at opposing conclusions.
- This study aimed to estimate the odds of painful sequelae among women with FGM/C compared to women without FGM/C using pooled odds ratios with adjustment for study design.

**What did the researchers do and find?:**

- We systematically searched and critically reviewed the literature documenting specific indicators of pain and obstetric complications after FGM/C. This included gynecologic outcomes such as dyspareunia (i.e., pain with intercourse or pain with vaginal penetration), dysuria (i.e., pain, burning, or discomfort with urination), dysmenorrhea (i.e., painful menstruation), and urinary tract infection, as well as obstetric outcomes such as prolonged labor, perineal tears, episiotomy (i.e., a surgical incision of the perineum allowing a newborn to pass through), cesarean section, and instrumental delivery.
- We identified 116 studies with a total of 77,324 women who had undergone FGM/C and 63,949 women without FGM/C.
- We found that pooled estimates from cross-sectional studies provide evidence that individuals with FGM/C are at higher risk for dyspareunia, dysuria, perineal tears, and prolonged labor. Pooled estimates from case–control studies additionally provide evidence that individuals with FGM/C are at higher risk for episiotomies. There was insufficient evidence to conclude that FGM/C is associated with dysmenorrhea, urinary tract infection, instrumental deliveries, or cesarean sections.

**What do these findings mean?:**

- As violent global conflict accelerates and the numbers of displaced individuals increases, clinicians globally should familiarize themselves with the complex clinical presentation and medical sequelae of women who have experienced FGM/C.
- Data from this study indicate that clinicians may want to consider the possibility of painful gynecological outcomes among patients who have undergone FGM/C and be aware of the potential for painful obstetrical sequelae.
- This study contributes to a severely neglected area of research that is becoming increasingly relevant and important to patient wellbeing.
- Our findings should be interpreted with caution because of several limitations of our work, including the use of numerous studies of low to medium quality, less than half of the primary studies appropriately accounting for confounding, and the use of all types of FGM/C in the meta-analysis.

## Introduction

**Fig 1:**
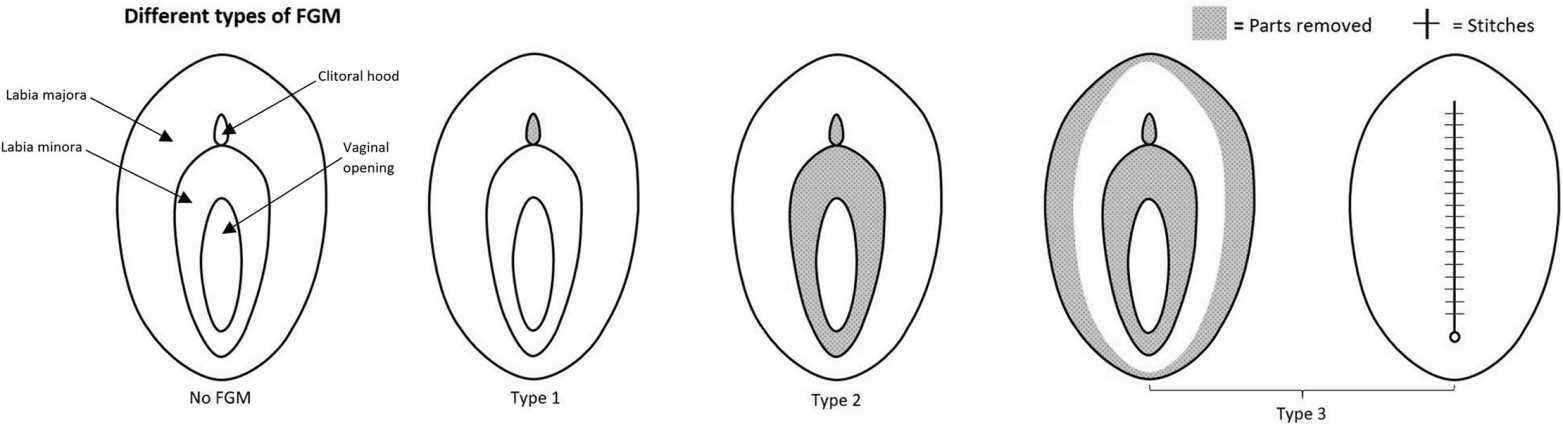
WHO classification of FGM/C. Type I FGM/C consists of partial or total removal of the clitoris and its prepuce, Type II consists of the removal of the clitoris and labia minora, and Type III consists of narrowing the vaginal orifice. FGM/C, female genital mutilation/cutting; WHO, World Health Organization.

Global statistics indicate that at least 200 million women and girls in 30 countries have undergone female genital mutilation/cutting (FGM/C) [1]. Approximately 70 million girls aged 0–14 years have been cut or may be at risk of genital cutting [2]. The World Health Organization (WHO) has categorized FGM/C severity into 4 degrees: Type I, or clitoridectomy, which consists of partial or total removal of the clitoris and its prepuce; Type II, or excision, which consists of the removal of the clitoris and labia minora; Type III, the most severe form, which is known as infibulation and consists of narrowing the vaginal orifice; and Type IV, which includes “all other harmful procedures to the female genitalia for non-medical purposes,” such as “pricking, pulling, piercing, incising, scraping and cauterization” [3]. The 4 types of FGM/C are described in Fig 1 [4].

As migration of forcibly displaced individuals increases, healthcare providers must familiarize themselves with the complicated clinical presentation and management of women and girls who have experienced FGM/C. According to the United Nations High Commission for Refugees, nearly 300,000 females from FGM/C-practicing countries applied for asylum in the European Union from 2013 to 2017. In addition, the percentage of asylum applicants from FGM/C-practicing countries increased from 6% to 9% of all applicants in 2013 and from 19% to 28% of all female applicants in 2017. In the same year, the top 4 countries of origin for female asylum seekers in the European Union included Iraq, Nigeria, Eritrea, and Somalia, and these countries represent over two-thirds of the total number of women applying for asylum in the European Union from FGM/C-practicing countries [5]. It is estimated that FGM/C Type I and II account for approximately 85% of all cases globally. In Iraq and Nigeria, for example, this is by far the most common kind of FGM/C [6]. However, in Eritrea and Somalia, FGM/C Type III is much more common, occurring in 38.5% and 79.3% of the population, respectively. While all types of FGM/C hold health implications, Type III, being the most severe form, is particularly concerning [7,8].

Health professionals often do not clinically recognize FGM/C or understand the negative health consequences associated with the practice; physicians in high-income countries are unfamiliar and uncomfortable treating patients with FGM/C [3,9]. Women who have undergone FGM/C themselves fear that practitioners do not have sufficient training to provide appropriate care [10]. There is a global need for increased physician education regarding FGM/C [11,12]. Further, there is a substantial need for research on the acute and chronic complications of FGM/C, their prevalence and manifestation, and guidance on treatment and management [13]. There are very few comprehensive systematic reviews documenting the presence of long-term complications following FGM/C [14–16], and a robust understanding of painful outcomes is elusive. Research in this area is both uncommon and important to patient care.

To address the gap in our understanding of the painful sequelae and obstetric complications of FGM/C, we performed a comprehensive systematic review and meta-analysis of the existing literature through December 19, 2019.

## Methods

We performed this systematic review and meta-analysis according to the Preferred Reporting Items for Systematic Reviews and Meta-Analyses (PRISMA) guidelines, as delineated in S1 Text [17].

### Search strategy

**Fig 2:**
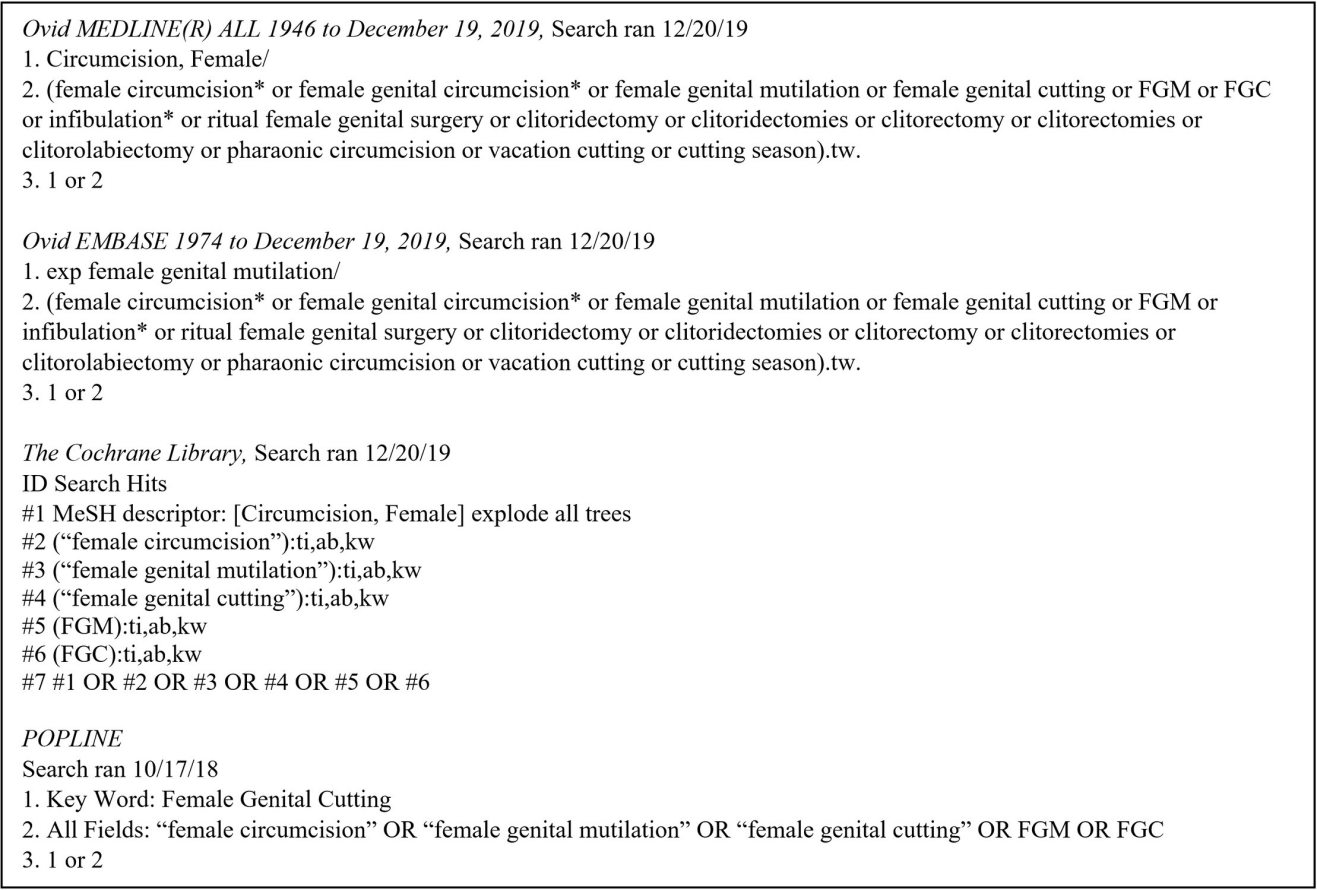
Search strategy employed to identify relevant studies. FGM/C, female genital mutilation/cutting.

A medical librarian (DD) performed a comprehensive literature search in multiple electronic databases, including Ovid MEDLINE, Ovid EMBASE, and The Cochrane Library (Wiley), from inception to December 19, 2019 for mentions of FGM/C. Additionally, a comprehensive literature search of POPLINE was conducted from inception to October 17, 2018, prior to its retirement. There were no restrictions on study type, language, or publication date. Bibliographies of articles that met inclusion criteria and papers citing included articles were retrieved by using the “View references” and “Cited by” features in Scopus. Full electronic database names and search strategies are included in Fig 2. Systematic reviews were not included in the meta-analysis, although their bibliographies were retrieved and assessed for inclusion.

### Inclusion and exclusion

Inclusion criteria included all studies describing the incidence or prevalence of painful sequelae associated with FGM/C. There were no limitations placed on age, ethnicity, nationality, or other characteristics. Articles published in a language other than English were included, and translations of these manuscripts were performed with readily available online translation tools. Exclusion criteria included articles that did not contain a clear indication of FGM/C as classified by WHO [18], articles in which pain measurements were unclear or absent, data that were not published in peer-reviewed medical journal articles (for example, presentations and abstracts), articles that were duplicates (in which case the first article to be published was included and the other excluded), review articles for which references were already used, and articles whose citations were found but for which the articles could not be located. Pain indicators of interest were determined through consultations with a physician expert in pain medicine. Primary outcomes included the following: 1) wound infection, urinary tract infection, or other infection associated with FGM/C; 2) abscess formation; 3) dyspareunia (i.e., pain with intercourse or pain with vaginal penetration); 4) gangrene or necrotizing fasciitis (i.e., the worsening of an infection that causes the death of soft tissues); 5) keloid or hypertrophic scar formation (i.e. fibrous lesions causing pain, itching, and decreasing skin compliance); 6) neuroma formation (i.e., the development of a painful nerve outgrowth); 7) dysuria (i.e., pain, burning, or discomfort with urination); 8) dysmenorrhea (i.e., painful menstruation) or hematocolpos (i.e., a painful accumulation of blood within the vagina); and 9) pregnancy and birth complications such as lacerations, episiotomy (i.e., a surgical incision of the perineum allowing a newborn to pass through), or cesarean section.

### Data extraction and quality appraisal

Two reviewers (JL and AW) screened manuscript titles and abstracts for inclusion and meticulous examination. A third reviewer (SH) made the final decision when discrepancy arose between the 2 primary reviewers. Two reviewers (JL and AW) then assessed full texts of articles for inclusion and meta-analysis. A third reviewer (SH) again made the decision on inclusion when discrepancies arose. Data were extracted by 2 reviewers (JL and AW) into an electronic database. These data were cross-checked, and discrepancies were resolved by a third reviewer (SH). Such data included study design, first author, data published, article title, journal title, number of participants with FGM/C, and prevalence of participants experiencing the predetermined pain symptoms.

The quality of included studies was independently assessed via validated appraisal instruments by 2 reviewers (JL and AW), and a third reviewer (SH) settled any disputes. Cross-sectional studies were evaluated by the Agency for Healthcare Research and Quality (AHRQ) Methodology Checklist [19], and case–control and cohort studies were evaluated with the Critical Appraisal Skills Programme (CASP) Checklist [20]. These appraisal tools accounted for confounding, among other measures of risk of bias and quality. The full appraisal tools and their items relating to bias and quality are presented in S3 Text. Case reports and case series were not appraised.

### Statistical analysis

Studies were grouped by design (cross-sectional, case–control, cohort, and case report), and case report studies were excluded from pooled analyses. Groups with fewer than 3 studies reporting a particular outcome were considered an inadequate body of literature for meta-analysis and were not included in pooled estimates. Pooled proportions describing pain sequelae among women with FGM/C were calculated from cross-sectional studies using random effects models. Associations between FGM/C and obstetric and pain outcomes of interest were examined using pooled odds ratios (ORs) for binary outcomes and standardized mean differences (SMDs) for continuous outcomes. Inverse variance weighting with Hartung–Knapp correction was used to calculate all pooled estimates.

Subgroup analysis was performed to assess and adjust for heterogeneity introduced by study design. Random effects models were used to pool estimates within and across subgroups. Two-tailed *t* tests were used to assess pooled and within-group effects, and chi-squared tests were used to assess for differences by study design.

Heterogeneity was assessed using an *I*^2^ statistic for all pooled analyses, where <70% was considered mild, 70%–90% was considered moderate, and >90% was considered substantial. For outcomes for which subgroup analysis was performed, residual heterogeneity was calculated to assess remaining heterogeneity after accounting for subgroup differences. All *p*-values were two-sided, and statistical significance was assessed at the 0.05-alpha level. We calculated 95% confidence intervals (CIs) to assess the precision of effect estimates on averages. For pooled estimates, 95% prediction intervals (95% PIs) were calculated to assess the precision of individual-level predictions. All analyses were performed in R Version 3.6.1 using the “meta” package [21].

## Results

**Fig 3:**
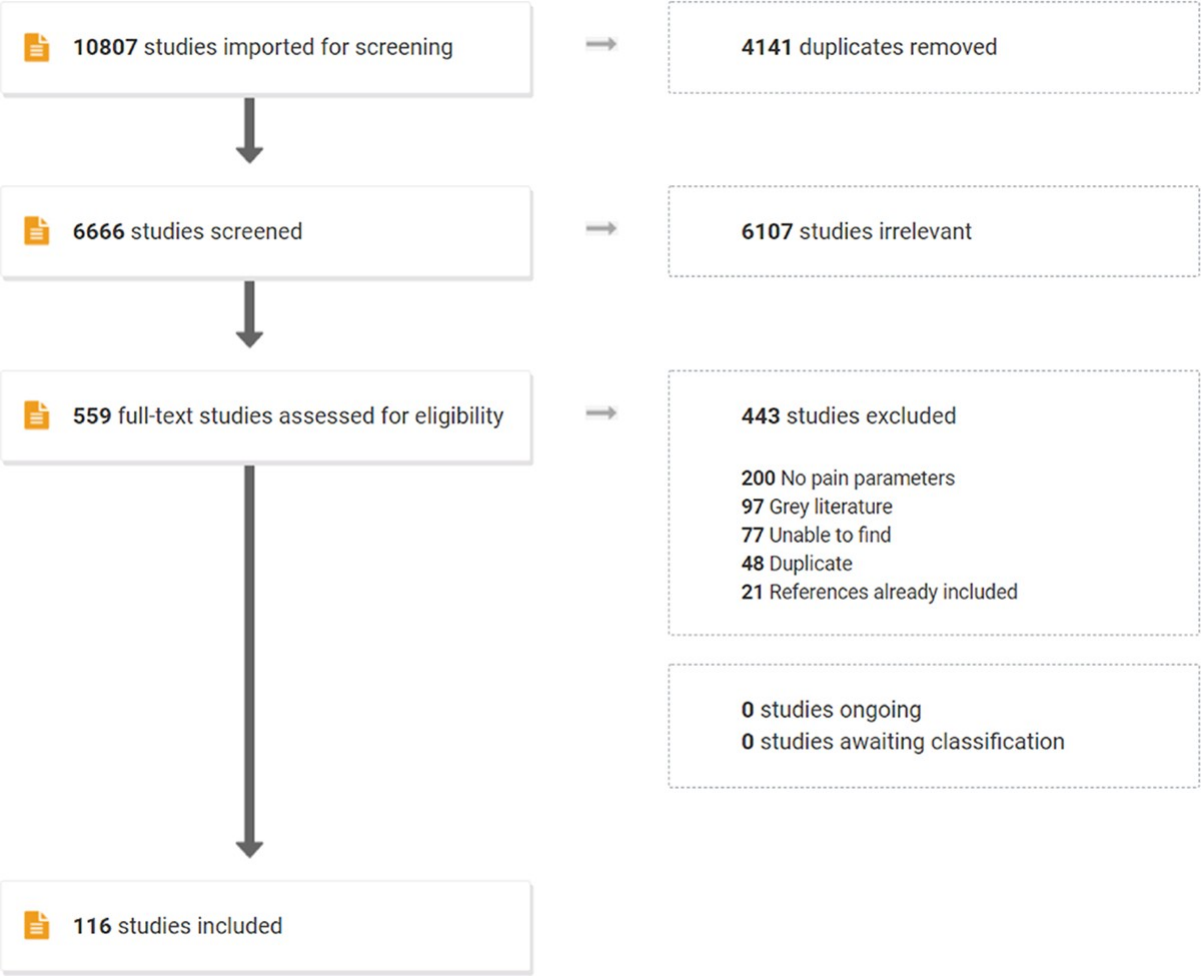
PRISMA flowchart. PRISMA, Preferred Reporting Items for Systematic Reviews and Meta-Analyses.

We identified 10,807 abstracts, which included 559 full-text articles that were assessed for eligibility. Of these, 443 studies were excluded because no quantitative data on pain parameters were presented (200) or they were unpublished works such as abstracts or conference proceedings (97), duplicates (48), unable to be located (77), or they included repeat data (21). 111 studies met inclusion criteria, which included 77,324 women who had undergone FGM/C and 63,949 women without FGM/C. The details of our systematic review are encapsulated by the PRISMA flowchart in Fig 3. In addition, S2 Text presents the characteristics of our included studies. We included 60 cross-sectional studies, 11 case–control studies, 8 cohort studies, and 37 case reports or case series. Further study characteristics, including quality and bias risk assessments, are shown in S3 Text, and citations for all included studies are in S4 Text.

**Fig 4:**
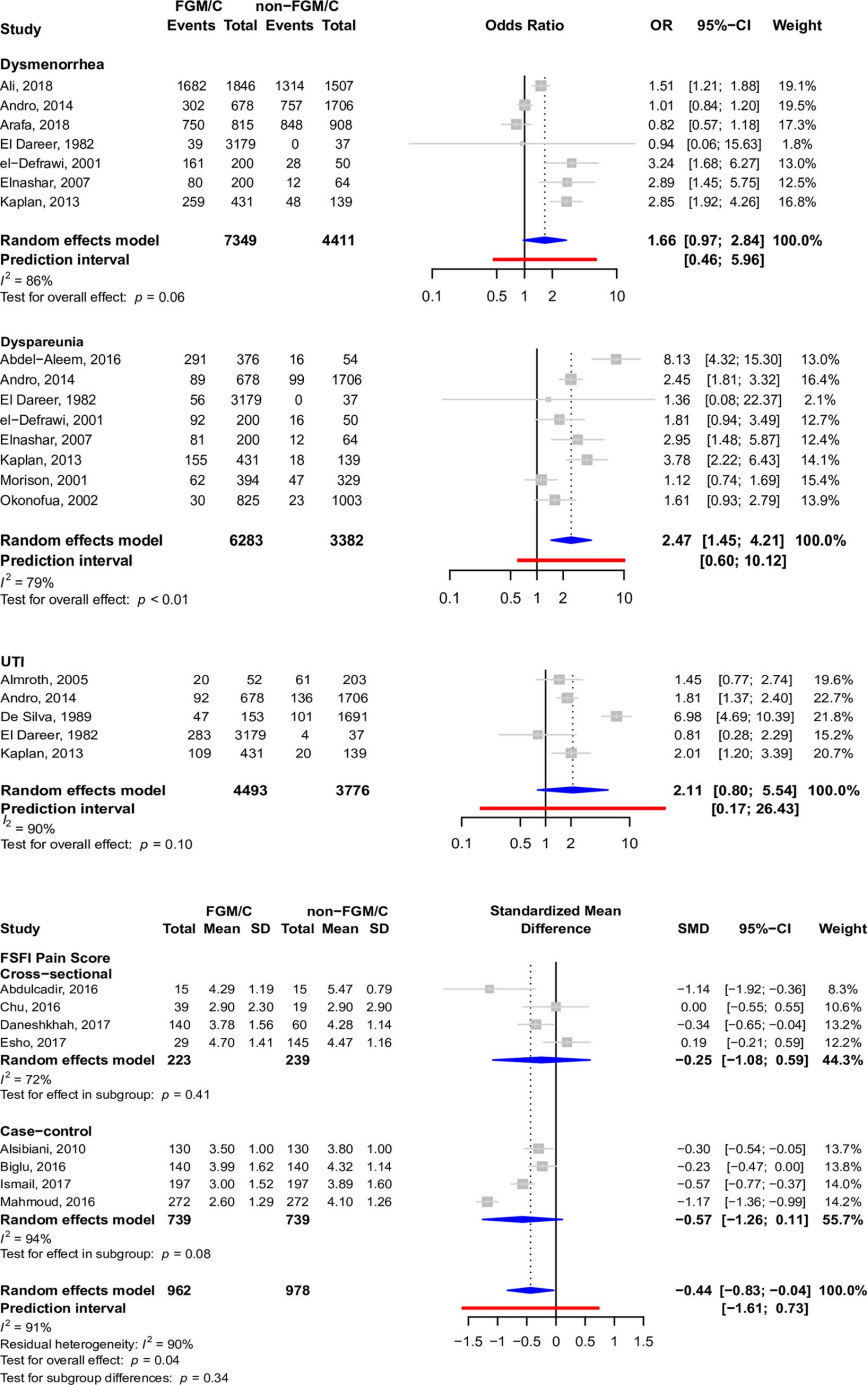
Forest plots depicting meta-analyses of dysmenorrhea, dyspareunia, UTI, and FSFI pain score in the setting of FGM/C. *t* tests were used to assess pooled and within-group effects, and chi-square tests were used to assess for differences by study design. CI, confidence interval; FGM/C, female genital mutilation/cutting; FSFI, Female Sexual Function Index; OR, odds ratio; SD, Standard Deviation; SMD, standardized mean difference; UTI, Urinary Tract Infection.

**Fig 5:**
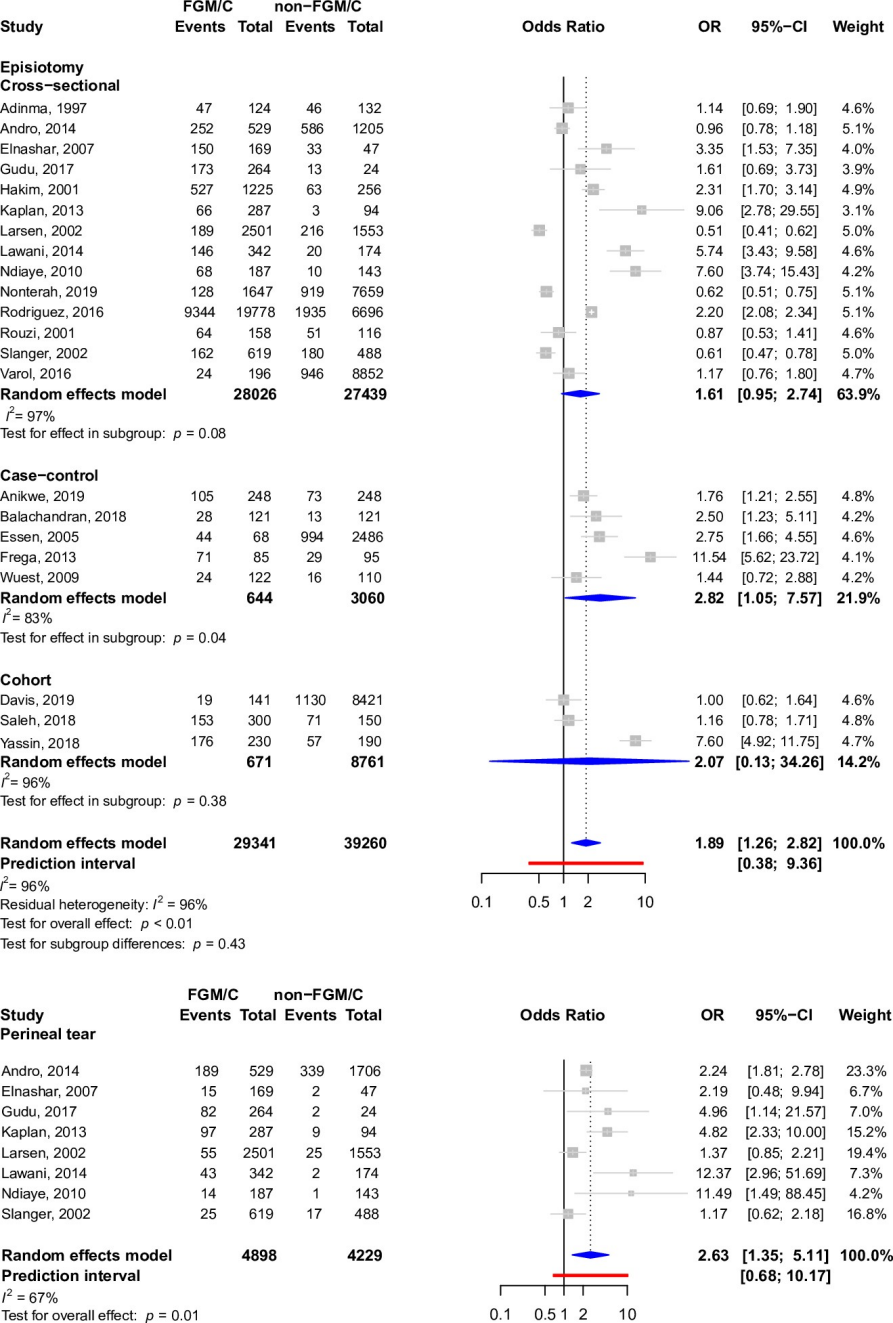
Forest plots depicting meta-analyses of episiotomy and perineal tears in the setting of FGM/C. *t* tests were used to assess pooled and within-group effects, and chi-squared tests were used to assess for differences by study design. CI, confidence interval; FGM/C, female genital mutilation/cutting; OR, odds ratio.

**Fig 6:**
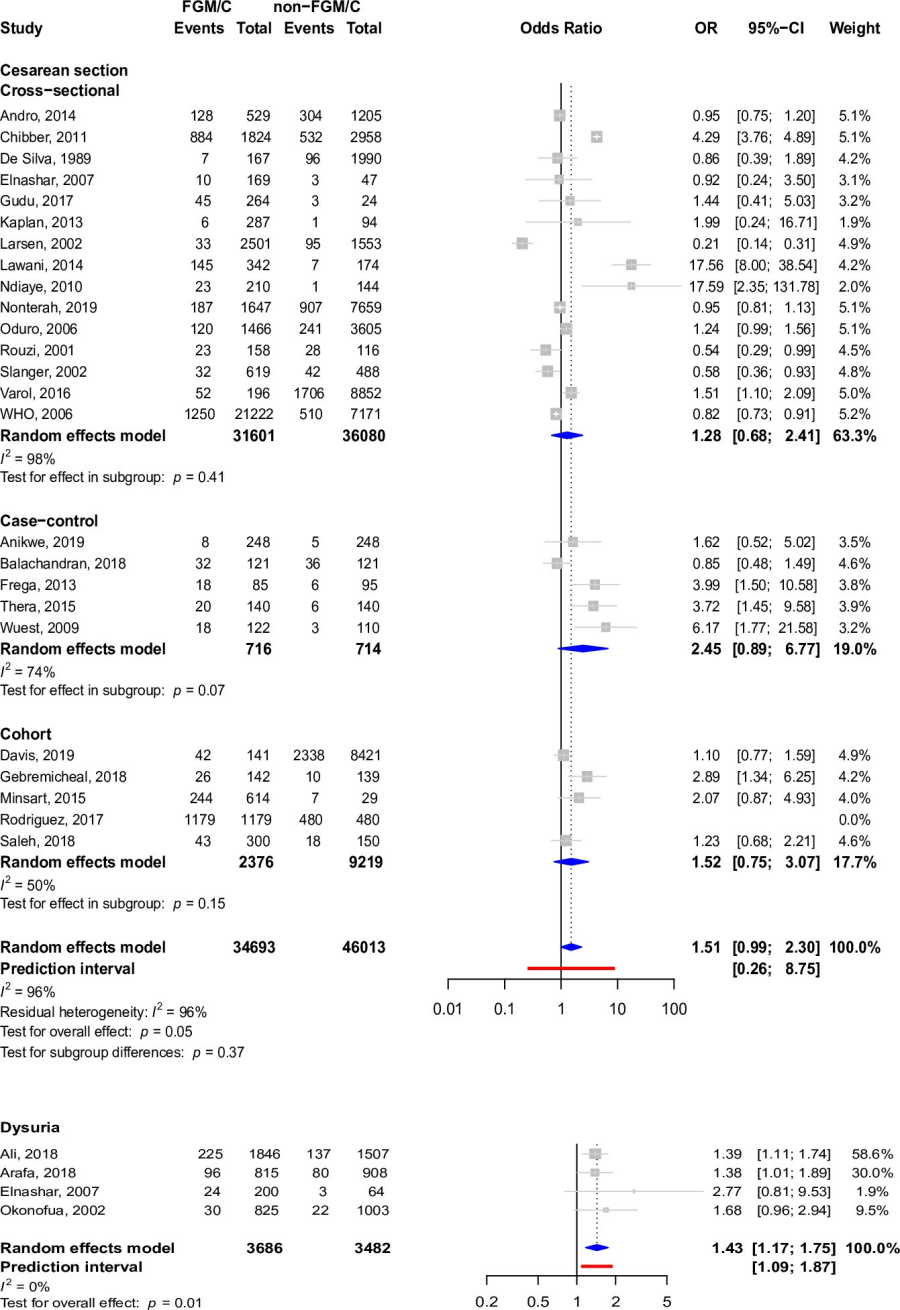
Forest plots depicting meta-analyses of cesarean delivery and dysuria in the setting of FGM/C. *t* tests were used to assess pooled and within-group effects, and chi-squared tests were used to assess for differences by study design. CI, confidence interval; FGM/C, female genital mutilation/cutting; OR, odds ratio.

**Fig 7:**
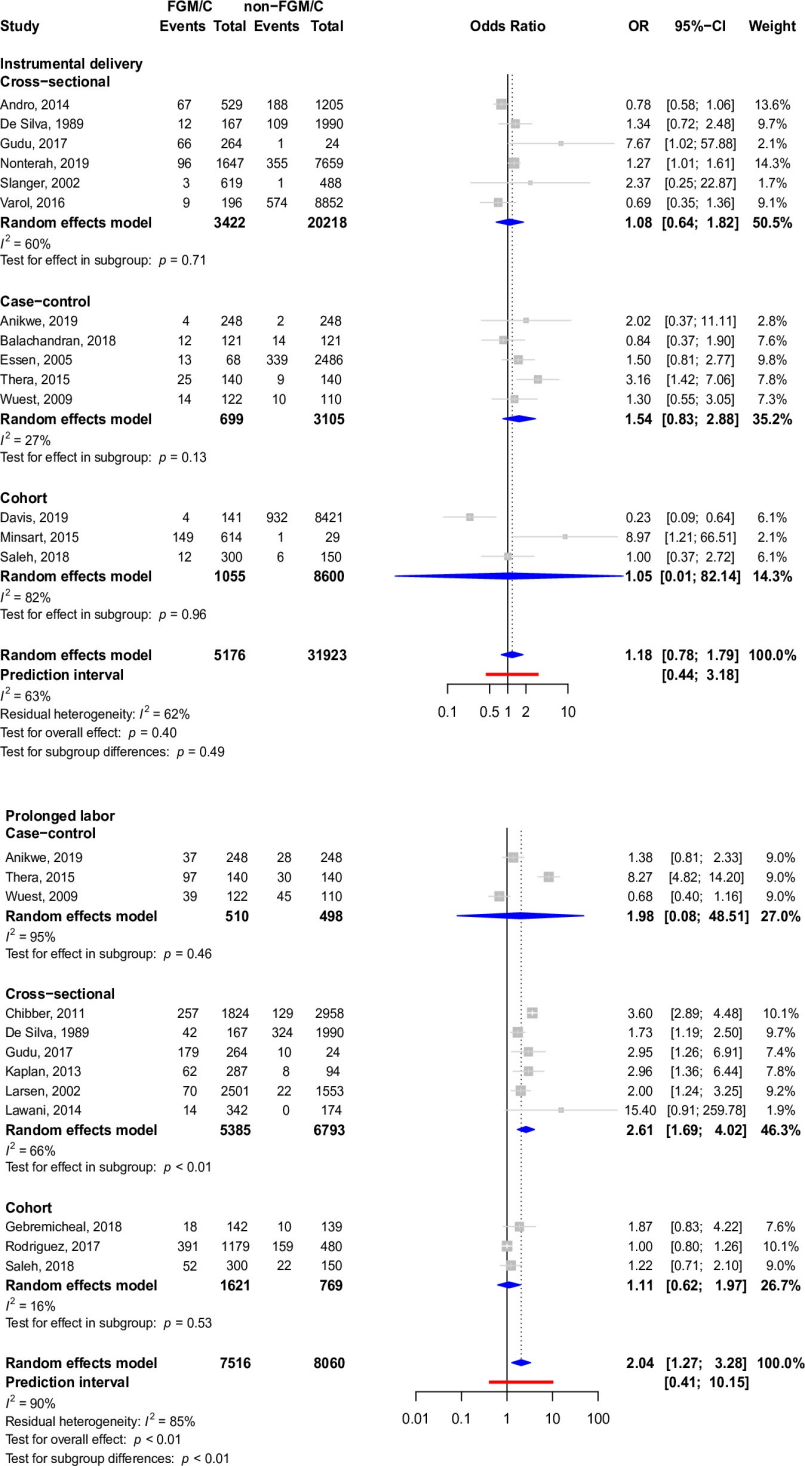
Forest plots depicting meta-analyses of instrumental delivery and prolonged labor in the setting of FGM/C. *t* tests were used to assess pooled and within-group effects, and chi-squared tests were used to assess for differences by study design. FGM/C, female genital mutilation/cutting; CI, confidence interval; OR, odds ratio.

The most widely described outcome in cross-sectional studies, cohort studies, case–control studies, and case reports in participants who had undergone FGM/C was cesarean section (4,889 participants in 42 studies). Other prominent outcomes included episiotomy (12,592 participants in 38 studies), instrumental delivery (535 participants in 20 studies), prolonged labor (1,434 participants in 20 studies), perineal tears (1,257 participants in 19 studies), and dyspareunia (1,955 participants in 39 studies), as well as indications of the Female Sexual Function Index (FSFI) pain score, which is a quantitative pain score that measures dyspareunia (962 participants in 8 studies), dysmenorrhea (4,017 participants in 26 studies), urinary tract infection (790 participants in 15 studies), and dysuria (697 participants in 18 studies). Forest plots depicting pooled associations between FGM/C and these outcomes are present in Figs 4–7. Other less common outcomes and their pooled prevalences are shown in Table 1. Studies typically focused on one or a few pain indicators, although some studies presented data regarding several of the pain outcomes.

**Table 1:** Pooled prevalences of obstetric and pain outcomes among women who have experienced FGM/C.

Pain Outcome	Number of Studies Used in Analysis	Pooled Proportion of FGM/C Participants Experiencing Event	95% CI	95% PI	*I*^2^	Contributing Studies
Short-term pain after procedure	5	0.69	(0.06, 0.99)	(0.00, 1.00)	99	Chalmers [22]; Momoh [23]; Mukoro [24]; Plo [25]; Sayed [26]
Wound or pelvic infection	12	0.12	(0.08, 0.19)	(0.02, 0.55)	97	Abor [27]; Bjälkander [28]; Bogale [29]; Chalmers [22]; Chu [30]; Dirie [31]; Dirie [32]; El Dareer [33] Gudu [34]; Kaplan [35]; Knight [36]; Momoh [23]
Lacerations	3	0.03	(0.00, 0.40)	(0.00, 1.00)	95	Nonterah [37]; Raouf [38]; Rouzi [39]
Intense pain (acute)	3	0.40	(0.07, 0.85)	(0.00, 1.00)	98	Abor [27]; Andro [40]; Dare [41]
Defibulation	7	0.93	(0.48, 0.99)	(0.05, 1.00)	97	Almroth [42]; Dirie [31]; Knight [36]; Nour [43]; Paliwal [44]; Raouf [38]; Rouzi [39]
Chronic pelvic pain	3	0.23	(0.07, 0.56)	(0.00, 0.99)	74	El-Defrawi [45]; Kaplan [46]; Zurynski [47]

**Abbreviations:** CI, confidence interval; FGM/C, female genital mutilation/cutting; PI, prediction interval.

FGM/C was associated with FSFI pain score (pooled SMD: −0.44, 95% CI: −0.83 to −0.04, *I*^2^: 91%, *p*-value = 0.04), dyspareunia (pooled OR: 2.47, 95% CI: 1.45–4.21, *I*^2^: 79%, *p*-value < 0.01), dysuria (pooled OR: 1.43, 95% CI: 1.17–1.75, *I*^2^: 0%, *p*-value = 0.01), perineal tears (pooled OR: 2.63, 95% CI: 1.35–5.11, *I*^2^: 67%, *p*-value = 0.01), episiotomy (pooled OR: 1.89, 95% CI: 1.26–2.82, *I*^2^: 96%, *p*-value < 0.01), and prolonged labor (pooled OR: 2.04, 95% CI: 1.27–3.28, *I*^2^: 90%, *p*-value < 0.01) on pooled analysis after adjustment for study design, if relevant. This can be seen in Figs 4–7. There was insufficient evidence from pooled analyses with adjustment for study design (when applicable) to conclude that there are associations between FGM/C and dysmenorrhea (pooled OR: 1.66, 95% CI: 0.97–2.84, *I*^2^: 86%, *p*-value = 0.06), urinary tract infection (pooled OR: 2.11, 95% CI: 0.80–5.54, *I*^2^: 90%, *p*-value = 0.10), cesarean section (pooled OR: 1.51, 95% CI: 0.99–2.30, *I*^2^: 96%, *p*-value = 0.05), or instrumental delivery (pooled OR: 1.18, 95% CI: 0.78–1.79, *I*^2^: 63%, *p*-value = 0.40). This can be seen in Figs 4, 6 and 7.

Effect sizes for prolonged labor (Fig 7) differed significantly by study design (*p*-value < 0.01), and residual heterogeneity (*I*^2^: 85%) was somewhat decreased compared to overall heterogeneity (*I*^2^: 90%). Within the cross-sectional design subgroup, women with FGM/C were two and a half times more likely to experience prolonged labor (pooled OR: 2.61, 95% CI: 1.69‒4.02, *I*^2^: 66%, p-value: <0.01). Although pooled analyses for case–control studies suggest that women with FGM/C were twice as likely to experience prolonged labor, the effect was unstable (pooled OR: 1.98, 95% CI: 0.08–48.51, *I*^2^: 95%, *p*-value = 0.46). Evidence from analyses pooled from cohort studies was insufficient to support the association.

There was no difference in effect size by study design for FSFI pain score (Fig 4), episiotomy (Fig 5), cesarean delivery (Fig 6), or instrumental delivery (Fig 7), and residual heterogeneity was not substantially decreased compared to overall heterogeneity for these outcomes. In general, the largest pooled effects sizes were from case–control studies, with the exception of prolonged labor, for which pooled estimates from cross-sectional studies had the largest magnitude (Fig 7).

Although differences in effect size by study design were not detected at a 0.05-alpha level for episiotomy (Fig 5), it is notable that while pooled estimates from case–control studies suggest that women with FGM/C are nearly 3 times as likely to require episiotomy, pooled estimates from cross-sectional designs suggest that women with FGM/C are only 60% more likely to require episiotomy. Moreover, the association was unstable in the cross-sectional subgroup.

The most common sources of potential bias (S3 Text) among the cross-sectional studies included not fully listing inclusion and exclusion criteria, not indicating whether or not participants were consecutive, not describing any assessments undertaken for quality assurance purposes such as test/retest of primary outcome measurements, not explaining participant exclusions from the analysis, and not mentioning how confounding was assessed and controlled. In addition, it was difficult to glean whether evaluators of subjective components of studies were masked to other aspects of the status of participants and how missing data were handled in the analyses in nearly all cross-sectional studies. For the 11 case–control studies, no study adequately addressed confounding factors, although all studies met the other CASP checklist criteria. All 8 cohort studies met CASP checklist criteria. Case reports and case series were not systematically assessed in this manner and were generally low-quality.

## Discussion

Women are affected by FGM/C in at least 28 African countries [48,49], with some also affected in the Middle Eastern and Southeast Asian countries of Yemen, Iraq, Indonesia, and Malaysia [13]. Every year, over 2 million girls experience FGM/C; in several countries such as Egypt, Eritrea, Guinea, Somalia, Mali, and Sierra Leone, over 90% of women have undergone FGM/C [50]. As forced displacement increases, providers in host nations must familiarize themselves with FGM/C, its consequences, and its management. While the immediate complications of FGM/C such as hemorrhage, immediate pain, shock, sepsis, swelling, bleeding, and tetanus are well-documented [12,14,51–55], the long-term pain and obstetric effects are less studied, and multiple studies have noted the need for additional research [13,51,52]. These complications, such as chronic pelvic pain, dysmenorrhea, and dyspareunia—which are described in this study—are relevant to practitioners globally, who are increasingly likely to encounter patients with FGM/C because of amplified migration [9,13,56,57].

Here, we present the most comprehensive review of pain and obstetric complications of FGM/C to our knowledge to date. Previous research has included systematic reviews and meta-analyses on the obstetric sequelae (such as prolonged labor and perineal tears) [58] and gynecological consequences of FGM/C (such as dyspareunia and urinary tract infections) [59], the most recent of which was Berg and colleagues in 2012 [60]. In the past decade, several relevant studies have been published, prompting the need for an updated aggregation that utilizes the additional precision of a growing body of literature. Furthermore, we examine several outcomes that have not previously been seen in FGM/C meta-analyses; specifically, we estimate the pooled effects of FGM/C on FSFI pain score, a validated method to measure dyspareunia, and we present pooled prevalence estimates for chronic pelvic pain and defibulation in the setting of FGM/C.

The present investigation identifies several pain and obstetric complications that are statistically associated with FGM/C on pooled analysis after adjustment for study design, including FSFI pain score, dyspareunia, dysuria, perineal tearing, prolonged labor, and episiotomy. Although outcomes such as dysmenorrhea, UTI, cesarean section, and instrumental delivery were not statistically associated with FGM/C, it is important to note that all of the pooled analyses presented here suffered from inadequate statistical power because of small sample sizes and high variability across studies. It is our opinion that the statistical significance of the pooled effect sizes is rather beside the point; the real value of the present study is the presentation of effect estimates and precision intervals derived from data aggregated across an entire body of literature that provide exploratory insights into the true location and variation of these population parameters.

The current meta-analysis incorporates more than one-and-a-half times as many studies as Berg and colleagues [60], allowing us to add precision and refine previous pooled estimates. Notably, the Berg and colleagues team had insufficient evidence to identify a significant association between FGM/C and perineal tears (pooled OR: 1.39; 95% CI: 0.99–1.95; *I*^2^: 55%; *p*-value = 0.08). Using numerous additional studies, we were able to achieve the precision needed for statistical significance, and our data suggest that women with FGM/C are more than twice as likely to experience prolonged labor, an effect that is considerably stronger than that identified by Berg and colleagues. In addition, we provide effect and precision estimates for FSFI pain score, a validated method to measure dyspareunia.

Almost all of our meta-analyses had high variability as assessed by the *I*^2^ statistic, and, although study design clearly accounted for some of the heterogeneity in the pooled estimate for prolonged labor, study design did not appear to account for a substantial portion of heterogeneity in pooled estimates related to FSFI pain score, episiotomy, cesarean section, or instrumental delivery. Nonetheless, we encourage adjustments that carefully consider study design, since our analyses are likely underpowered to detect differences in effect sizes by study design.

Type of FGM/C is a likely source of heterogeneity. In our meta-analysis of perineal tearing, 2 of the studies with the highest ORs (Lawani and colleagues [61] and Gudu [34]) had study samples that included large proportions (72% and 100%, respectively) of women with Type II and Type III FGM/C (i.e., more severe forms). Studies reporting lower ORs such as Slanger and colleagues (2002) [62] and Larsen and colleagues (2002) [63] had larger proportions of women with Type I FGM/C (72% and 71%, respectively). However, not all studies in this analysis presented stratified analyses by type of FGM/C (such as Andro and colleagues [40] and Elnashar and colleagues [64]), making further adjustment by this confounder impossible in the present study. However, it is likely that the type of FGM/C contributes to heterogeneity in pooled analyses of other outcomes.

Geographic focus is likely to be another source of heterogeneity. For example, in pooled analyses of episiotomy, of the 22 contributing studies, only 4 focused on populations in western Africa: Frega and colleagues (2013) [65], which focused on Burkina Faso; Kaplan and colleagues (2013) [46], which focused on The Gambia; Ndiaye and colleagues (2010) [66], which focused on Burkina Faso; and Nonterah and colleagues (2019) [37], which focused on Ghana. These studies contributed some of the largest effect sizes. The remaining studies focused on eastern African countries such as Nigeria, Ethiopia, Somalia, Egypt, and Sudan or focused on participants from numerous countries. It is possible that there are differences in medical and cultural practices relating to episiotomy across geographic region.

Interview setting is also likely to contribute to study heterogeneity. In the pooled analysis for dysmenorrhea, the 3 studies with the largest effect sizes (el-Defrawi and colleagues [2001] [45], Elnashar and colleagues [2007] [64], and Kaplan and colleagues [2013] [46]) interviewed patients in medical settings such as family planning centers, obstetric and gynecologic clinics, and other hospital settings, and studies reporting smaller effect sizes took place in nonmedical settings. For example, Ali and colleagues (2018) [67] interviewed participants in their homes, and Arafa and colleagues (2018) [68] surveyed university students.

Physician knowledge regarding effective diagnosis and management of patients with FGM/C in high-income nations is lacking. A study conducted in the United Kingdom with 618 physicians affiliated with the Royal College of Obstetricians and Gynaecologists (RCOG) found that only 25% felt as though they had received sufficient training to treat patients with FGM/C [69]. Physicians were also unfamiliar with guidelines concerning pregnancy in FGM/C, and less than one-third of doctors were aware that defibulation during pregnancy is recommended at approximately 20 weeks’ gestation. In addition, over half were unaware how to contact and refer patients to specialist services, and 25% were unaware of the association between FGM/C and pelvic infection. Another study in which 8 gynecologists in Sweden were interviewed about their experiences following the delivery of patients with FGM/C found that most of the physicians were unaware of any associations between FGM/C and neonatal distress [70]. Such gaps in knowledge can lead to adverse outcomes.

Evidence also exists that women having undergone FGM/C experience disrespect from medical professionals who are unfamiliar with the practice, potentially threatening doctor–patient relationships and outcomes. A survey of 432 Somali refugees giving birth in Canada after FGM/C found that the vast majority reported offensive comments from their caregivers because of their cutting. Patients reported being regarded with disgust and shock [10]. The majority also felt that doctors did not understand their pain and that physicians did not understand that women with FGM/C experienced particularly severe postpartum pain. Two out of five women stated that they would not return to the same hospital for future deliveries, and more than 10% of women stated that they would prefer to not attend any hospital for future births, a particularly poor outcome given the potential birthing complications in women with FGM/C. Others have additionally noted that cultural insensitivity and ignorance regarding FGM/C among physicians in high-income nations deters these women from seeking future medical care, leading to unquantifiable future morbidity [13]. The literature also documents women who have been affected by FGM/C being left in stirrups for extended periods of time as medical students and residents inspect their anatomy, and these patients reported feeling dehumanized when interacting with healthcare providers in high-income nations [71]. These reports indicate that a poor understanding of FGM/C among healthcare professionals can lead to significant health ramifications for this vulnerable patient population.

Our systematic review and meta-analysis were conducted according to PRISMA guidelines, followed a registered protocol (CRD42018115848), and utilized validated tools to assess risk of bias and study quality. This study is an important contribution to the understanding of the painful and obstetric outcomes of FGM/C. This investigation had several limitations. Many of the included studies have limited methodological development and low validity, and this is a common issue regarding literature documenting FGM/C [72,73]. Less than half of the primary studies appropriately accounted for confounding, such as type of FGM/C, and this presents a limitation of the current systematic review. Our findings were also limited by the inclusion of all types of FGM/C in the meta-analysis, as information pertaining to the specific types of FGM/C as dictated by WHO was oftentimes unclear or absent. Manifestations of FGM/C oftentimes do not fit neatly into WHO’s limited categories [50,52]. Critics note that these WHO categories provide a false framework that envision distinct forms of FGM/C, but in practice, forms of FGM/C are too nuanced and complex for these 4 groupings. Forms of FGM/C can significantly differ even within these categories, and FGM/C varies extensively by region and by practitioner. Some authors have also noted that interpretations of these categories vary widely, causing ethical and legal quandaries in the reporting of FGM/C [9]. With this understanding and because of the variation in reporting, this study did not make distinctions between types of FGM/C and sought to capture pain outcomes associated with all forms of the practice.

Data from this study indicate that clinicians globally should be prepared to provide appropriate treatment and screening to patients who have undergone FGM/C while considering the possibility of painful gynecological outcomes. Physicians should also be prepared for the possibility of painful and complex obstetrical sequelae. This work contributes to a neglected area of research that is becoming increasingly relevant and important to patient wellbeing. Investigations into appropriate and adequate treatment, particularly in the obstetric population, are warranted. Obstetricians ought to consider the risks that women with FGM/C face, such as an increased likelihood of perineal tears and episiotomy, and these care teams should formulate appropriate clinical management strategies to best serve this population.

## Supporting information

S1 Text PRISMA checklist. PRISMA, Preferred Reporting Items for Systematic Reviews and Meta-Analyses
(DOC)

S2 Text Included study characteristics. (DOCX)

S3 Text Quality and bias risk assessment. (DOCX)

S4 Text Included study citations. (DOCX)

## Abbreviations

AHRQ: Agency for Healthcare Research and Quality
CASP: Critical Appraisal Skills Programme
CI: confidence interval
FGM/C: female genital mutilation/cutting
FSFI: Female Sexual Function Index
OR: odds ratio
PI: prediction interval
PRISMA: Preferred Reporting Items for Systematic Reviews and Meta-Analyses
RCOG: Royal College of Obstetricians and Gynaecologists
SMD: standardized mean difference
WHO: World Health Organization
