# Supplementary material for: Painful gynecologic and obstetric complications of female genital mutilation/cutting: A systematic review and meta-analysis
Source: PLoS Med. 2020 Mar 31;17(3):e1003088. doi: 10.1371/journal.pmed.1003088 (PMC7108709; doi:10.1371/journal.pmed.1003088)
Supplement: S2 Text — (DOCX) [file pmed.1003088.s002.docx]

| **Author** | **Date Published** | **Study Design** | **Country of origin for FGM/C population** | **Host country** | **FGM/C elicited via examination or patient report?** | **Type of FGM/C discussed** | **Control group country of origin (same as FGM/C population or different?):** | **Total number FGM/C** | **Total number Non-FGM/C** |
| --- | --- | --- | --- | --- | --- | --- | --- | --- | --- |
| Abdel-Aleem | 2016 | Cross-sectional | Egypt | Same | Exam | Described, not WHO type | Same | 376 | 54 |
| Abdulcadir | 2016 | Cross-sectional | Multiple: Eritrea, Somalia, Ivory Coast, Guiney, Burkina Faso, Mali, Senegal | Switzerland | Exam | IIb, IIIa,b | Europe, South America, Africa | 15 | 15 |
| Abdulcadir | 2017 | Case report/series | Ivory Coast | Switzerland | Exam | IIc | - | 1 |  |
| Abdulcadir | 2013 | Case report/series | Somalia | Switzerland | Exam | III | - | 1 |  |
| Abdulcadir | 2016 | Case report/series | Multiple: Guinea, Ethiopia, Sudan, Burkina Faso, Egypt, Yemen, Ivory Coast, Djibouti, Liberia, Senegal, Cameroun, Gambia, Mali, Sierra Leone | Switzerland | Exam | I, II, III | - | 129 |  |
| Abdulcadir | 2012 | Case report/series | Somalia | Switzerland | Exam | III | - | 1 |  |
| Abor | 2006 | Cross-sectional | Ghana | Same | Self-report | Unclear, maybe I/II | - | 34 |  |
| Adetoro | 1986 | Case report/series | Nigeria | Same | Exam | Unclear, maybe I/II | - | 1 |  |
| Adinma | 1997 | Cross-sectional | Nigeria | Same | Exam | Described, likely I/II | Same | 124 | 132 |
| Akotionga | 2001 | Cross-sectional | Burkina Faso | Same | Exam | Mostly III | - | 49 |  |
| Al-Hussaini | 2003 | Cross-sectional | Egypt | Same | Exam | I, II | - | 254 |  |
| Albert | 2015 | Cross-sectional | Unclear | United Kingdom | Exam | III | - | 94 |  |
| Ali | 2018 | Cross-sectional | Egypt | Same | Self-report | Not assessed | Same | 1846 | 1507 |
| Almroth-Berggren | 2001 | Cross-sectional | Sudan | Same | Self-report | Described, nearly all Type III | - | 60 |  |
| Almroth | 2005 | Cross-sectional | Sudan | Same | Exam | Described, likely III/II | Same | 52 | 203 |
| Alsibiani | 2010 | Case-control | Saudi Arabia | Same | Self-report | I, II, III | Same | 130 | 130 |
| Anand | 2014 | Case report/series | Somalia | USA | Exam | III | - | 1 |  |
| Andro | 2014 | Cross-sectional | Multiple African countries | France | Self-report | Described, maybe I, II, III | France and Africa | 678 | 1706 |
| Anikwe | 2019 | Case-control | Nigeria | Same | Exam | I, II, III | Same | 248 | 248 |
| Arafa | 2018 | Cross-sectional | Egypt | Egypt | Self-report | Unclear | Same | 815 | 908 |
| Arbesman | 1993 | Case report/series | Somalia | USA | Self-report | Unclear | - | 12 |  |
| Asuen | 1977 | Case report/series | Nigeria | Same | Exam | Described, likely II/III | - | 2 |  |
| Aziem-Abdallah-Ali | 2011 | Case report/series | Sudan | Same | Exam | Type III | - | 1 |  |
| Baker | 1993 | Case report/series | Sudan | USA | Exam | Described, likely I/II | - | 1 |  |
| Balachandran | 2018 | Case-control | Multiple | United Kingdom | Exam | I, II, III | Same | 121 | 121 |
| Biglu | 2016 | Case-control | Iran | Same | Exam | I | Same | 140 | 140 |
| Birge | 2017 | Cross-sectional | Sudan | Same | Exam | I, II, III | Same | 210 | 29 |
| Birge | 2017 | Case report/series | Sudan | Same | Exam | I, II, III | - | 27 |  |
| Bjalkander | 2012 | Cross-sectional | Sierra Leone | Same | Self-report | Unclear | - | 258 |  |
| Bogale | 2014 | Cross-sectional | Ethiopia | Same | Self-report | I, II, III | Same | 486 | 133 |
| Brisson | 2001 | Case report/series | "African" country | USA | Exam | Described, likely III | - | 1 |  |
| Chalmers | 2005 | Cross-sectional | Somalia | Canada | Self-report | Mostly III, some I/II and unknown | - | 432 |  |
| Chen | 2004 | Case report/series | Sudan | USA | Exam | III | - | 1 |  |
| Chibber | 2011 | Cross-sectional | Kuwait | Same | Exam | I, II, III | Same | 1824 | 2958 |
| Chu | 2016 | Cross-sectional | Multiple: Gambia, Guinea, Mali, Sierra Leone | USA | Self-report | I, II, III | Same | 46 | 68 |
| Craven | 2016 | Case report/series | Morocco | USA | Exam | III | - | 1 |  |
| Daneshkhah | 2017 | Cross-sectional | Iran | Same | Self-report and occasional exams to clarify | Unclear, but maybe I/II/III | Same | 140 | 60 |
| Dare | 2004 | Cross-sectional | Nigeria | Same | Exam | I, II | Same | 522 |  |
| Davis | 2019 | Cohort | Multiple: Somalia, Sudan, Sierra Leone, Ethiopia, Egypt, Indonesia, others | Australia | Exam | I, II, III | European and African | 141 | 8421 |
| Daw | 1970 | Case report/series | Sudan | Same | Exam | Unclear, likely I/II | - | 3 |  |
| De Silva | 1989 | Cross-sectional | Multiple: Sudan, Eritrea, Somalia, Egypt | Same | Exam | I/II/III | Same | 167 | 1990 |
| Dewhurst | 1964 | Case report/series | Sudan | United Kingdom | Exam | Unclear, likely III | - | 1 |  |
| Dilbaz | 2019 | Case report/series | Somalia | Turkey | Exam | III | - | 1 |  |
| Diouf | 2017 | Case report/series | Senegal | Same | Exam | II, III | - | 14 |  |
| Dirie | 1992 | Cross-sectional | Somalia | Same | Exam | Unclear | - | 290 |  |
| Dirie | 1991 | Cross-sectional | Somalia | Same | Exam | I/II/III | - | 118 |  |
| Dorflinger | 2000 | Cross-sectional | Sudan | Same | Exam | III | - | 39 |  |
| Egwuatu | 1981 | Case report/series | Nigeria | Same | Exam | Unclear | - | 58 |  |
| El-Agwany | 2015 | Case report/series | Egypt | Same | Exam | II | - | 1 |  |
| El Dareer | 1982 | Cross-sectional | Sudan | Same | Exam | III, likely I, II | Same | 3179 | 37 |
| el-Defrawi | 2001 | Cross-sectional | Egypt | Same | Exam | Described, likely I/II | Same | 200 | 50 |
| Elnashar | 2007 | Cross-sectional | Egypt | Same | Self-report | Unclear | Same | 200 | 64 |
| Epstein | 2001 | Case report/series | Somalia | USA | Exam | III | - | 1 |  |
| Erian | 1995 | Case report/series | Somalia | Australia | Exam | III | - | 3 |  |
| Esho | 2017 | Cross-sectional | Kenya | Same | Self-report | Unclear | Same | 202 | 145 |
| Essen | 2005 | Case-control | Ethiopia, Somalia | Sweden | Exam | Mostly III, some I/II | European and African | 68 | 2486 |
| Fernandez-Aguilar | 2003 | Case report/series | Guinea | Belgium | Exam | III | - | 1 |  |
| Foldes | 2006 | Cohort | Unclear | France | Exam | II, III | - | 453 |  |
| Frega | 2013 | Cross-sectional | Burkina Faso | Same | Exam | I, II | Same | 85 | 95 |
| Gebremicheal | 2018 | Cohort | Ethiopia | Same | Exam | I, II, III | Same | 142 | 139 |
| Gudu | 2014 | Case report/series | Ethiopia | Same | Exam | III | - | 1 |  |
| Gudu | 2017 | Cross-sectional | Ethiopia | Same | Exam | III mostly, some II | Same | 264 | 24 |
| Gultekin | 2016 | Case report/series | "African countries" | Turkey | Exam | III | - | 2 |  |
| Hadid | 2015 | Case report/series | Ivory Coast | Canada | Exam | III | - | 1 |  |
| Hakim | 2001 | Cross-sectional | Ethiopia | Same | Exam | I, II, III | Same | 1225 | 256 |
| Hanly | 1995 | Case report/series | Saudi Arabia | Same | Exam | Described, likely III | - | 10 |  |
| Ismail | 2017 | Case-control | Egypt | Same | Exam | I, II | Same | 197 | 197 |
| Jones | 1999 | Cross-sectional | Burkina Faso, Mali | Same | Exam | I, II, III | - | 1468 |  |
| Kaplan | 2013 | Cross-sectional | Gambia | Same | Exam | I, II, III | Same | 431 | 139 |
| Kaplan | 2011 | Cross-sectional | Gambia | Same | Exam | I, II, III | - | 871 |  |
| Klouman | 2005 | Cross-sectional | Tanzania | Same | Exam | I, II, III | - | 396 |  |
| Knight | 1999 | Cross-sectional | Somalia, Ethiopia, Eritrea, Djibouti | Australia | Exam | I, II, III | - | 51 |  |
| Larsen | 2002 | Cross-sectional | Nigeria | Same | Exam | I, II | Same | 2501 | 1553 |
| Lawani | 2014 | Cross-sectional | Nigeria | Same | Exam | I, II, III | Same | 342 | 174 |
| Mahmoud | 2016 | Case-control | Egypt | Same | Self-report | I, II, III | Same | 272 | 272 |
| Mawad | 1994 | Cross-sectional | Sudan | Same | Exam | Unclear, but maybe I/II/III | - | 934 |  |
| McCleary | 1994 | Case report/series | Canada | Somalia | Exam | III | - | 1 |  |
| McSwiney | 1992 | Case report/series | Somalia | United Kingdom | Exam | III | - | 1 |  |
| Minsart | 2015 | Cohort | Djibouti | Djibouti | Exam | I, II, III | Same | 614 | 29 |
| Momoh | 2001 | Cross-sectional | "Sub-Saharan Africa" | United Kingdom | Self-report | I, II, III | Same | 108 | 81 |
| Morison | 2001 | Cross-sectional | Gambia | Same | Exam | I, II, III | Same | 668 | 489 |
| Mukoro | 2004 | Cross-sectional | Nigeria | Same | Self-report | I, II | - | 46 |  |
| Ndiaye | 2010 | Cross-sectional | Burkina Faso | Same | Exam | I, II, III | Same | 210 | 144 |
| Nonterah | 2019 | Cross-sectional | Ghana | Same | Exam | Unclear | Same | 1647 | 7659 |
| Nour | 2006 | Case report/series | Somalia | USA | Exam | III | - | 1 |  |
| Nour | 2006 | Cross-sectional | Somalia, Sudan, Ethiopia | USA | Exam | III | - | 40 |  |
| Oduro | 2006 | Cross-sectional | Ghana | Same | Exam | Unclear | Same | 1466 | 3605 |
| Okonofua | 2002 | Cross-sectional | Nigeria | Same | Exam | I, II | Same | 825 | 1003 |
| Orji | 2006 | Cross-sectional | Nigeria | Same | Exam | I, II | Same | 423 | 77 |
| Paliwal | 2014 | Cross-sectional | Multiple: Somalia, Eritrea, Sudan, Yemen, Sierra Leone, Nigeria, Kenya | United Kingdom | Exam | III | - | 253 |  |
| Penna | 2002 | Cohort | Unclear | Italy | Exam | III | - | 25 |  |
| Pereda | 2012 | Case report/series | Mauritania | Spain | Exam | I, III | - | 3 |  |
| Plo | 2014 | Cross-sectional | Ivory Coast | Same | Exam | I, II, III | Same | 60 | 349 |
| Raouf | 2011 | Cross-sectional | Multiple: Somalia, Ethiopia, Gambia, Egypt, Kuwait | United Kingdom | Exam | III | - | 250 |  |
| Rodriguez | 2017 | Cohort | Multiple: Burkina Faso, Ghana, Kenya, Nigeria, Senegal, Sudan | Same | Exam | Unclear, but likely I, II, III | Same | 1179 | 480 |
| Rodriguez | 2016 | Cross-sectional | Multiple: Burkina Faso, Ghana, Kenya, Nigeria, Senegal, Sudan | Same | Exam | III | Same | 19778 | 6696 |
| Rouzi | 2012 | Case-control | Multiple: Sudan, Somalia, Ethiopia, Egypt, Yemen | Saudi Arabia | Exam | III | Same | 388 | 388 |
| Rouzi | 2001 | Cross-sectional | Sudan, Somalia | Same | Exam | III | Same | 158 | 116 |
| Rouzi | 2001 | Case report/series | Saudi Arabia | Same | Exam | I | - | 21 |  |
| Rouzi | 2017 | Cross-sectional | Sudan | Saudi Arabia | Exam | I, II, III | - | 107 |  |
| Rouzi | 2014 | Case report/series | Eritrea | Saudi Arabia | Exam | I | - | 1 |  |
| Saleh | 2018 | Cohort | Egypt | Same | Exam | I, II | Same | 300 | 150 |
| Sayed | 1996 | Cross-sectional | Egypt | Same | Self-report | Unclear | Same | 1074 | 658 |
| Schiotz | 2012 | Case report/series | “Africa” | Norway | Exam | I or II | - | 1 |  |
| Sharfi | 2013 | Cross-sectional | Sudan | Same | Exam | I, II, III | Same | 1468 | 532 |
| Slanger | 2002 | Cross-sectional | Nigeria | Same | Exam | I, II, III. IV | Same | 619 | 488 |
| Thera | 2015 | Case-control | Mali | Same | Exam | I, II, III | Same | 140 | 140 |
| Torky | 2018 | Case report/series | Egpyt | Same | Exam | I | - | 1 |  |
| Vangen | 2004 | Case report/series | Somalia | Norway | Self-report | III mostly, one I/II | - | 23 |  |
| Varol | 2016 | Cross-sectional | African countries, Middle East, Asia | Australia | Exam | I, II, III, IV | Australian, other | 196 | 8852 |
| WHO | 2006 | Cross-sectional | Multiple: Burkina Faso, Ghana, Kenya, Nigeria, Senegal, Sudan | Same | Exam | I, II, III | Same | 21222 | 7171 |
| Wuest | 2009 | Case-control | Multiple: Somalia, Sudan, Ethiopia, Tanzania, Kenya, Egypt, "Far East" | Switzerland | Exam | I, II, III, IV | Switzerland, other | 122 | 110 |
| Yassin | 2018 | Cohort | Sudan | Same | Exam | I, III | Same | 230 | 190 |
| Zayed | 2012 | Cross-sectional | Egypt | Same | Self-report | Unclear | Same | 156 | 88 |
| Zoorob | 2019 | Case report/series | Somalia | USA | Exam | III | - | 1 |  |
| Zurynski | 2017 | Cross-sectional | Multiple: Sudan, Malaysia, Indonesia, Kenya, Eritrea, Sierra Leone, Uganda, Somalia, Egypt, Australia | Australia | Exam | I, II, III, IV | - | 31 |  |
