# Supplementary material for: Painful gynecologic and obstetric complications of female genital mutilation/cutting: A systematic review and meta-analysis
Source: PLoS Med. 2020 Mar 31;17(3):e1003088. doi: 10.1371/journal.pmed.1003088 (PMC7108709; doi:10.1371/journal.pmed.1003088)
Supplement: S3 Text — (DOCX) [file pmed.1003088.s003.docx]

Quality assessment and risk of bias for included studies

Key

Low risk of bias/high quality

|  |  |
| --- | --- |

High risk of bias/low quality

|  |  |  |
| --- | --- | --- |

Unclear risk of bias/indeterminate quality

|  |  |
| --- | --- |

Cross Sectional Studies, assessed with ARHQ Methodology Checklist:

| **Author name** | **Year** | **1. Define the source of information (survey, record review)** | **2. List inclusion and exclusion criteria for exposed and unexposed subjects (cases and controls) or refer to previous publications** | **3. Indicate time period used for identifying patients** | **4. Indicate whether or not subjects were consecutive if not population-based** | **5. Indicate if evaluators of subjective components of study were masked to other aspects of the status of the participants** | **6. Describe any assessments undertaken for quality assurance purposes (e.g., test/retest of primary outcome measurements)** | **7. Explain any patient exclusions from analysis** | **8. Describe how confounding was assessed and/or controlled** | **9. If applicable, explain how missing data were handled in the analysis** | **10. Summarize patient response rates and completeness of data collection** | **11. Clarify what follow-up, if any, was expected and the percentage of patients for which incomplete data or follow-up was obtained** |
| --- | --- | --- | --- | --- | --- | --- | --- | --- | --- | --- | --- | --- |
| Abdel-Aleem | 2016 |  |  |  |  |  |  |  |  |  |  |  |
| Abor | 2006 |  |  |  |  |  |  |  |  |  |  |  |
| Adinma | 1997 |  |  |  |  |  |  |  |  |  |  |  |
| Akotionga | 2001 |  |  |  |  |  |  |  |  |  |  |  |
| Albert | 2015 |  |  |  |  |  |  |  |  |  |  |  |
| Al-Hussaini | 2003 |  |  |  |  |  |  |  |  |  |  |  |
| Ali | 2018 |  |  |  |  |  |  |  |  |  |  |  |
| Almroth-Berggren | 2001 |  |  |  |  |  |  |  |  |  |  |  |
| Almroth | 2005 |  |  |  |  |  |  |  |  |  |  |  |
| Andro | 2014 |  |  |  |  |  |  |  |  |  |  |  |
| Arafa | 2018 |  |  |  |  |  |  |  |  |  |  |  |
| Abdulcadir | 2016 |  |  |  |  |  |  |  |  |  |  |  |
| Birge | 2017 |  |  |  |  |  |  |  |  |  |  |  |
| Bjalkander | 2012 |  |  |  |  |  |  |  |  |  |  |  |
| Bogale | 2014 |  |  |  |  |  |  |  |  |  |  |  |
| Chalmers | 2005 |  |  |  |  |  |  |  |  |  |  |  |
| Chibber | 2011 |  |  |  |  |  |  |  |  |  |  |  |
| Chu | 2016 |  |  |  |  |  |  |  |  |  |  |  |
| Daneshkhah | 2017 |  |  |  |  |  |  |  |  |  |  |  |
| Dare | 2004 |  |  |  |  |  |  |  |  |  |  |  |
| De Silva | 1989 |  |  |  |  |  |  |  |  |  |  |  |
| Dirie | 1991 |  |  |  |  |  |  |  |  |  |  |  |
| Dirie | 1992 |  |  |  |  |  |  |  |  |  |  |  |
| Dorflinger | 2000 |  |  |  |  |  |  |  |  |  |  |  |
| El Dareer | 1982 |  |  |  |  |  |  |  |  |  |  |  |
| el-Defrawi | 2001 |  |  |  |  |  |  |  |  |  |  |  |
| Elnashar | 2007 |  |  |  |  |  |  |  |  |  |  |  |
| Esho | 2017 |  |  |  |  |  |  |  |  |  |  |  |
| Gudu | 2014 |  |  |  |  |  |  |  |  |  |  |  |
| Hakim | 2001 |  |  |  |  |  |  |  |  |  |  |  |
| Jones | 1999 |  |  |  |  |  |  |  |  |  |  |  |
| Kaplan | 2011 |  |  |  |  |  |  |  |  |  |  |  |
| Kaplan | 2013 |  |  |  |  |  |  |  |  |  |  |  |
| Klouman | 2005 |  |  |  |  |  |  |  |  |  |  |  |
| Knight | 1999 |  |  |  |  |  |  |  |  |  |  |  |
| Larsen | 2002 |  |  |  |  |  |  |  |  |  |  |  |
| Lawani | 2014 |  |  |  |  |  |  |  |  |  |  |  |
| Mawad | 1994 |  |  |  |  |  |  |  |  |  |  |  |
| Momoh | 2001 |  |  |  |  |  |  |  |  |  |  |  |
| Morison | 2001 |  |  |  |  |  |  |  |  |  |  |  |
| Mukoro | 2004 |  |  |  |  |  |  |  |  |  |  |  |
| Ndiaye | 2010 |  |  |  |  |  |  |  |  |  |  |  |
| Nonterah | 2019 |  |  |  |  |  |  |  |  |  |  |  |
| Nour | 2006 |  |  |  |  |  |  |  |  |  |  |  |
| Oduro | 2006 |  |  |  |  |  |  |  |  |  |  |  |
| Okonofu | 2002 |  |  |  |  |  |  |  |  |  |  |  |
| Orji | 2006 |  |  |  |  |  |  |  |  |  |  |  |
| Paliwal | 2014 |  |  |  |  |  |  |  |  |  |  |  |
| Plo | 2014 |  |  |  |  |  |  |  |  |  |  |  |
| Raouf | 2011 |  |  |  |  |  |  |  |  |  |  |  |
| Rodriguez | 2016 |  |  |  |  |  |  |  |  |  |  |  |
| Rouzi | 2001 |  |  |  |  |  |  |  |  |  |  |  |
| Rouzi | 2017 |  |  |  |  |  |  |  |  |  |  |  |
| Sayed | 1996 |  |  |  |  |  |  |  |  |  |  |  |
| Sharfi | 2013 |  |  |  |  |  |  |  |  |  |  |  |
| Slanger | 2002 |  |  |  |  |  |  |  |  |  |  |  |
| Varol | 2016 |  |  |  |  |  |  |  |  |  |  |  |
| WHO | 2006 |  |  |  |  |  |  |  |  |  |  |  |
| Zayed | 2012 |  |  |  |  |  |  |  |  |  |  |  |
| Zurynski | 2017 |  |  |  |  |  |  |  |  |  |  |  |

Case-Control studies, assessed with CASP checklist (2014):

| **Author** | **Year** | **1. Did the study address a clearly focused issue** | **2. Did the authors use an appropriate method to answer their question** | **3. Were the cases recruited in an acceptable way** | **4.  Were the controls selected in an acceptable way** | **5. Was the exposure accurately measured to minimize bias** | **6. (a) What confounding factors have the authors accounted for** | **6.  (b) Have the authors taken account of the potential confounding factors in the design and/or in their analysis** |
| --- | --- | --- | --- | --- | --- | --- | --- | --- |
| Alsibiani | 2010 |  |  |  |  |  |  |  |
| Anikwe | 2019 |  |  |  |  |  |  |  |
| Balachandran | 2018 |  |  |  |  |  |  |  |
| Biglu | 2016 |  |  |  |  |  |  |  |
| Essen | 2005 |  |  |  |  |  |  |  |
| Frega | 2013 |  |  |  |  |  |  |  |
| Ismail | 2017 |  |  |  |  |  |  |  |
| Mahmoud | 2016 |  |  |  |  |  |  |  |
| Rouzi | 2012 |  |  |  |  |  |  |  |
| Thera | 2015 |  |  |  |  |  |  |  |
| Wuest | 2009 |  |  |  |  |  |  |  |

Cohort studies, CASP checklist (2014):

| **Author** | **Year** | **1. Did the study address a clearly focused issue** | **2. Was the cohort recruited in an acceptable way** | **3. Was the exposure accurately measured to minimize bias** | **4. Was the outcome accurately measured to minimize bias** | **5. (a) Have the authors identified all important confounding factors** | **5. (b) Have they taken account of the confounding factors in the design and/or analysis** | **6. (a) Was the follow up of subjects complete enough** | **6. (b) Was the follow up of subjects long enough** |
| --- | --- | --- | --- | --- | --- | --- | --- | --- | --- |
| Davis | 2019 |  |  |  |  |  |  |  |  |
| Foldes | 2006 |  |  |  |  |  |  |  |  |
| Gebremicheal | 2018 |  |  |  |  |  |  |  |  |
| Minsart | 2015 |  |  |  |  |  |  |  |  |
| Penna | 2002 |  |  |  |  |  |  |  |  |
| Rodriguez | 2017 |  |  |  |  |  |  |  |  |
| Saleh | 2018 |  |  |  |  |  |  |  |  |
| Yassin | 2018 |  |  |  |  |  |  |  |  |
