# Supplementary material for: Painful gynecologic and obstetric complications of female genital mutilation/cutting: A systematic review and meta-analysis
Source: PLoS Med. 2020 Mar 31;17(3):e1003088. doi: 10.1371/journal.pmed.1003088 (PMC7108709; doi:10.1371/journal.pmed.1003088)
Supplement: S4 Text — (DOCX) [file pmed.1003088.s004.docx]

1. Abdel-Aleem MA, Elkady MM, Hilmy YA. The relationship between female genital cutting and sexual problems experienced in the first two months of marriage. International Journal of Gynecology & Obstetrics. 2016;132(3): 305-8.

2. Abdulcadir J, Demicheli FB, Willame A, Recordon N, Petignat P. Posttraumatic stress disorder relapse and clitoral reconstruction after female genital mutilation. Obstetrics & Gynecology. 2017;129(2): 371-6.

3. Abdulcadir J, Botsikas D, Bolmont M, Bilancioni A, Djema DA, Demicheli FB, et al. Sexual anatomy and function in women with and without genital mutilation: a cross-sectional study. The Journal of Sexual Medicine. 2016;13(2): 226-37.

4. Abdulcadir J, Dällenbach P. Overactive bladder after female genital mutilation/cutting (FGM/C) type III. BMJ Case Reports. 2013: 1-4.

5. Abdulcadir J, Dugerdil A, Yaron M, Irion O, Boulvain M. Obstetric care of women with female genital mutilation attending a specialized clinic in a tertiary center. International Journal of Gynecology & Obstetrics. 2016;132(2): 174-8.

6. Abdulcadir J, Pusztaszeri M, Vilarino R, Dubuisson JB, Vlastos AT. Clitoral neuroma after female genital mutilation/cutting: a rare but possible event. The Journal of Sexual Medicine. 2012;9(4): 1220-5.

7. Abor PA. Female genital mutilation: Psychological and reproductive health consequences. The case of Kayoro traditional area in Ghana. Gender and Behaviour. 2006;4(1): 659-84.

8. Adetoro OO, Ebomoyi E. Health implications of traditional female circumcision in pregnancy. Asia‐Oceania Journal of Obstetrics and Gynaecology. 1986;12(4): 489-92.

9. Adinma J. Current status of female circumcision among Nigerian Igbos. West African Journal of Medicine. 1997;16(4): 227-31.

10. Akotionga M, Traore O, Lakoande J, Kone B. External genital excision sequelae at the Yalgado Ouedraogo national central hospital: epidemiology and surgical treatment. Gynecologie, Obstetrique & Fertilite. 2001;29(4): 295-300.

11. Albert J, Bailey E, Duaso M. Does the timing of deinfibulation for women with type 3 female genital mutilation affect labour outcomes? British Journal of Midwifery. 2015;23(6): 430-7.

12. Al-Hussaini TK. Female genital cutting: types, motives and perineal damage in laboring Egyptian women. Medical Principles and Practice. 2003;12(2): 123-8.

13. Ali HAAEW, Arafa AE, Allah NAEFA, Fahim AS. Prevalence of Female Circumcision among Young Women in Beni-Suef, Egypt: A Cross-Sectional Study. Journal of Pediatric and Adolescent Gynecology. 2018;31(6): 571-4.

14. Almroth L, Bedri H, El Musharaf S, Satti A, Idris T, Hashim MSK, et al. Urogenital complications among girls with genital mutilation: a hospital-based study in Khartoum. African Journal of Reproductive Health. 2005: 118-24.

15. Almroth-Berggren LA, Staffan Bergström, Osman Mahmoud Hassanein, Nagla El Hadi, Ulla-Britt Lithell, Vanja. Reinfibulation among women in a rural area in central Sudan. Health Care for Women International. 2001;22(8): 711-21.

16. Alsibiani SA, Rouzi AA. Sexual function in women with female genital mutilation. Fertility and Sterility. 2010;93(3): 722-4.

17. Anand M, Stanhope TJ, Occhino JA. Female genital mutilation reversal: a general approach. International Urogynecology Journal. 2014;25(7): 985-6.

18. Andro A, Cambois E, Lesclingand M. Long-term consequences of female genital mutilation in a European context: Self perceived health of FGM women compared to non-FGM women. Social Science & Medicine. 2014;106: 177-84.

19. Anikwe CC, Ejikeme BN, Obiechina NJ, Okorochukwu BC, Obuna JA, Onu FA, et al. Female genital mutilation and obstetric outcome: A cross-sectional comparative study in a tertiary hospital in Abakaliki South East Nigeria. European Journal of Obstetrics & Gynecology and Reproductive Biology: X. 2019;1: 1-5.

20. Arafa AE, Elbahrawe RS, Shawky SM, Abbas AM. Epidemiological and gynecological correlates with female genital mutilation among Beni-Suef University students; cross sectional study. Middle East Fertility Society Journal. 2018;23(3): 184-8.

21. Arbesman M, Kahler L, Buck GM. Assessment of the impact of female circumcision on the gynecological, genitourinary and obstetrical health problems of women from Somalia: literature review and case series. Women & Health. 1993;20(3): 27-42.

22. Asuen M. Maternal septicaemia and death after circumcision. Tropical Doctor. 1977;7(4): 177-8.

23. Aziem-Abdallah-Ali A, Mohammed AA, Mohammed Ali AK. Large inclusion cyst complicating female genital mutilation. Clin Pract. 2011;1(4): e121.

24. Baker CA, Gilson GJ, Vill MD, Curet LB. Female circumcision: obstetric issues. American Journal of Obstetrics & Gynecology. 1993;169(6): 1616-8.

25. Balachandran AA, Duvalla S, Sultan AH, Thakar R. Are obstetric outcomes affected by female genital mutilation? International Urogynecology Journal. 2018;29(3): 339-44.

26. Biglu M-H, Farnam A, Abotalebi P, Biglu S, Ghavami M. Effect of female genital mutilation/cutting on sexual functions. Sexual & Reproductive Healthcare. 2016;10: 3-8.

27. Birge O, Arslan D, Ozbey E, Adiyeke M, Kayar I, Erkan M, et al. Which type of circumcision is more harmful to female sexual functions? Clinical and Experimental Obstetrics & Gynecology 2017;44(5): 691-4.

28. Birge O, Ozbey E, Arslan D, Adiyeke M, Demir F, Kayars I. Clitoral keloids after female genital circumcision in early age. Clincal and Experimental Obstetrics & Gynecology. 2017;44(4): 511-4.

29. Bjälkander O, Bangura L, Leigh B, Berggren V, Bergström S, Almroth L. Health complications of female genital mutilation in Sierra Leone. International Journal of Women's Health. 2012;4: 321.

30. Bogale D, Markos D, Kaso M. Prevalence of female genital mutilation and its effect on women’s health in Bale zone, Ethiopia: a cross-sectional study. BMC Public Health. 2014;14(1): 1076.

31. Brisson P, Patel H, Feins N. Female circumcision. Journal of Pediatric Surgery. 2001;36(7): 1068-9.

32. Chalmers B, Omer-Hashi K, Khosid H. Female Genital Mutilation and Obstetric Care. 1^st^ ed. Trafford Publishing, 2005; 1-114.

33. Chen G, Dharia SP, Steinkampf MP, Callison S. Infertility from female circumcision. Fertility and Sterility. 2004;81(6): 1692-4.

34. Chibber R, El-Saleh E, El Harmi J. Female circumcision: obstetrical and psychological sequelae continues unabated in the 21st century. The Journal of Maternal-Fetal & Neonatal Medicine. 2011;24(6): 833-6.

35. Chu T, Akinsulure-Smith AM. Health outcomes and attitudes toward female genital cutting in a community-based sample of West African immigrant women from high-prevalence countries in New York City. Journal of Aggression, Maltreatment & Trauma. 2016;25(1): 63-83.

36. Craven S, Kavanagh A, Khavari R. Female genital mutilation management in the ambulatory clinic setting: a case study and review of the literature. Journal of Surgical Case Reports. 2016;(6): 1-3.

37. Daneshkhah F, Allahverdipour H, Jahangiri L, Andreeva T. Sexual function, mental well-being and quality of life among Kurdish circumcised women in Iran. Iranian Journal of Public Health. 2017;46(9): 1265.

38. Dare F, Oboro V, Fadiora S, Orji E, Sule-Odu A, Olabode T. Female genital mutilation: an analysis of 522 cases in South-Western Nigeria. Journal of Obstetrics and Gynaecology. 2004;24(3): 281-3.

39. Davis G, Jellins J. Female genital mutilation: Obstetric outcomes in metropolitan Sydney. Australian and New Zealand Journal of Obstetrics and Gynaecology. 2019;59(2): 312-6.

40. Daw E. Female circumcision and infibulation complicating delivery. Practitioner. 1970(204): 559-63.

41. De Silva S. Obstetric sequelae of female circumcision. European Journal of Obstetrics & Gynecology and Reproductive Biology. 1989;32(3): 233-40.

42. Dewhurst CJ, Michelson A. Infibulation complicating pregnancy. British Medical Journal. 1964;2(5422): 1442.

43. Dilbaz B, İflazoğlu N, Tanın SA. An overview of female genital mutilation. Turkish Journal of Obstetrics and Gynecology. 2019 Jun;16(2): 129.

44. Diouf AA, Diallo M, Mbodj A, Gassama O, Guèye M, Moreau JC, et al. Surgical treatment of complication of female genital mutilation in Pikine Hospital, Senegal. African Journal of Reproductive Health. 2017;21(1): 122-5.

45. Dirie MA, Lindmark G. A hospital study of the complications of female circumcision. Tropical Doctor. 1991;21(4): 146-8.

46. Dirie M, Lindmark G. The risk of medical complications after female circumcision. East African Medical Journal. 1992;69(9): 479-82.

47. Dorflinger A, Kuhn P, Dreher E. Female circumcision-(Not) A purely African problem. Geburtshilfe und Frauenheilkunde. 2000;60(11): 531-5.

48. Egwuatu V, Agugua N. Complications of female circumcision in Nigerian Igbos. BJOG: An International Journal of Obstetrics & Gynaecology. 1981;88(11): 1090-3.

49. El Dareer A. Woman, why do you weep? Circumcision and its consequences: 57 Caledonian Road, London N1 9DN, UK; Zed Press; 1982.

50. El-agwany AS. A large clitoral epidermal dermoid cyst: A rare long term complication after female circumcision. Middle East Fertility Society Journal. 2015;20(1): 57-9.

51. El-Defrawi MH, Lotfy G, Dandash KF, Refaat AH, Eyada M. Female genital mutilation and its psychosexual impact. Journal of Sex & Marital Therapy. 2001;27(5): 465-73.

52. Elnashar A, Abdelhady R. The impact of female genital cutting on health of newly married women. International Journal of Gynecology & Obstetrics. 2007;97(3): 238-44.

53. Epstein D, Graham P, Rimsza M. Medical complications of female genital mutilation. Journal of American College Health. 2001;49(6): 275-80.

54. Erian MM, Goh JT. Female circumcision. Australian and New Zealand Journal of Obstetrics and Gynaecology. 1995;35(1): 83-5.

55. Esho T, Kimani S, Nyamongo I, Kimani V, Muniu S, Kigondu C, et al. The ‘heat’goes away: sexual disorders of married women with female genital mutilation/cutting in Kenya. Reproductive Health. 2017;14(1): 164.

56. Essén B, Sjöberg N-O, Gudmundsson S, Östergren P-O, Lindqvist PG. No association between female circumcision and prolonged labour: a case control study of immigrant women giving birth in Sweden. European Journal of Obstetrics & Gynecology and Reproductive Biology. 2005;121(2): 182-5.

57. Fernández-Aguilar S, Noël J-C. Neuroma of the clitoris after female genital cutting. Obstetrics & Gynecology. 2003;101(5): 1053-4.

58. Foldés P, Louis-Sylvestre C. Results of surgical clitoral repair after ritual excision: 453 cases. Gynecologie, Obstetrique & Fertilite. 2006;34(12): 1137-41.

59. Frega A, Puzio G, Maniglio P, Catalano A, Milazzo GN, Lombardi D, et al. Obstetric and neonatal outcomes of women with FGM I and II in San Camillo Hospital, Burkina Faso. Archives of Gynecology and Obstetrics. 2013;288(3): 513-9.

60. Gebremicheal K, Alemseged F, Ewunetu H, Tolossa D, Ma’alin A, Yewondwessen M, et al. Sequela of female genital mutilation on birth outcomes in Jijiga town, Ethiopian Somali region: a prospective cohort study. BMC Pregnancy and Childbirth. 2018;18(1): 305.

61. Gudu W. Acute vulvar pain in a lady with post circumcision inclusion cyst of the vulva containing stones: a case report. BMC Women's Health. 2014;14(1): 9002.

62. Gudu W, Abdulahi M. Labor, delivery, and postpartum complications in nulliparous women with female genital mutilation admitted to Karamara Hospital. Ethiopian Medical Journal. 2017;55(1): 11-7.

63. Gültekin İB, Altınboğa O, Dur R, Kara OF, Küçüközkan T. Surgical reconstruction in female genital mutilation. Turkish Journal of Urology. 2016;42(2): 111.

64. Hadid V, Dahan MH. A case of chronic abdominal neuropathic pain and burning after female genital cutting. Case Reports in Obstetrics and Gynecology. 2015: 1-4.

65. Hakin L. Impact of female genital mutilation on maternal and neonatal outcomes during parturition. East African Medical Journal. 2001;78(5): 255-8.

66. Hanly M, Ojeda V. Epidermal inclusion cysts of the clitoris as a complication of female circumcision and pharaonic infibulation. The Central African Journal of Medicine. 1995;41(1): 22-4.

67. Ismail SA, Abbas AM, Habib D, Morsy H, Saleh MA, Bahloul M. Effect of female genital mutilation/cutting; types I and II on sexual function: case-controlled study. Reproductive Health. 2017;14(1): 108.

68. Jones H, Diop N, Askew I, Kaboré I. Female genital cutting practices in Burkina Faso and Mali and their negative health outcomes. Studies in Family Planning. 1999;30(3): 219-30.

69. Kaplan A, Forbes M, Bonhoure I, Utzet M, Martín M, Manneh M, et al. Female genital mutilation/cutting in The Gambia: long-term health consequences and complications during delivery and for the newborn. International Journal of Women's Health. 2013;5: 323-31.

70. Kaplan A, Hechavarría S, Martín M, Bonhoure I. Health consequences of female genital mutilation/cutting in the Gambia, evidence into action. Reproductive Health. 2011;8(1): 26.

71. Klouman E, Manongi R, Klepp KI. Self‐reported and observed female genital cutting in rural Tanzania: associated demographic factors, HIV and sexually transmitted infections. Tropical Medicine & International Health. 2005;10(1): 105-15.

72. Knight R, Hotchin A, Bayly C, Grover S. Female Genital Mutilation‐Experience of The Royal Women's Hospital, Melbourne. Australian and New Zealand Journal of Obstetrics and Gynaecology. 1999;39(1): 50-4.

73. Larsen U, Okonofua FE. Female circumcision and obstetric complications. International Journal of Gynecology & Obstetrics. 2002;77(3): 255-65.

74. Lawani LO, Onyebuchi AK, Iyoke CA, Okeke NE. Female genital mutilation and efforts to achieve Millennium Development Goals 3, 4, and 5 in southeast Nigeria. International Journal of Gynecology & Obstetrics. 2014;125(2): 125-8.

75. Mahmoud MIH. Effect of female genital mutilation on female sexual function, Alexandria, Egypt. Alexandria Journal of Medicine. 2016;52(1): 55-9.

76. Mawad N, Hassanein O. Female circumcision: Three years' experience of common complications in patients treated in Khartoum teaching hospitals. Journal of Obstetrics and Gynaecology. 1994;14(1): 40-3.

77. McCleay PH. Female genital mutilation and childbirth: a case report. Birth. 1994;21(4): 221-3.

78. McSwiney M, Saunders P. Female circumcision: a risk factor in postpartum haemorrhage. Journal of Postgraduate Medicine. 1992;38(3): 136.

79. Minsart A-F, N'guyen T-S, Ali Hadji R, Caillet M. Maternal infibulation and obstetrical outcome in Djibouti. The Journal of Maternal-Fetal & Neonatal Medicine. 2015;28(14): 1741-6.

80. Momoh C, Ladhani S, Lochrie DP, Rymer J. Female genital mutilation: analysis of the first twelve months of a southeast London specialist clinic. BJOG: An International Journal of Obstetrics & Gynaecology. 2001;108(2): 186-91.

81. Morison L, Scherf C, Ekpo G, Paine K, West B, Coleman R, et al. The long‐term reproductive health consequences of female genital cutting in rural Gambia: a community‐based survey. Tropical Medicine & International Health. 2001;6(8): 643-53.

82. Mukoro U. A survey on the psychosexual implications of female genital mutilation on Urhobo women of the Niger Delta Communities of Nigeria. Journal of Human Ecology. 2004;16(2): 147-50.

83. Ndiaye P, Diongue M, Faye A, Ouedraogo D, Tal AD. Female genital mutilation and complications in childbirth in the province of Gourma (Burkina Faso). Sante Publique (Vandoeuvre-les-Nancy, France). 2010;22(5): 563-70.

84. Nonterah EA, Kanmiki EW, Agorinya IA, Sakeah E, Mariatu T, Juliana K, et al. Prevalence and adverse obstetric outcomes of female genital mutilation among women in rural Northern Ghana. European Journal of Public Health. 2019: 1-6.

85. Nour NM. Urinary calculus associated with female genital cutting. Obstetrics & Gynecology. 2006;107(2): 521-3.

86. Nour NM, Michels KB, Bryant AE. Defibulation to treat female genital cutting: effect on symptoms and sexual function. Obstetrics & Gynecology. 2006;108(1): 55-60.

87. Oduro A, Ansah P, Hodgson A, Afful T, Baiden F, Adongo P, et al. Trends in the prevalence of female genital muti-lation and its effect on delivery outcomes in the kassena-nankana district of northern Ghana. Ghana Medical Journal. 2006;40(3): 87-92.

88. Okonofua FE, Larsen U, Oronsaye F, Snow RC, Slanger T. The association between female genital cutting and correlates of sexual and gynaecological morbidity in Edo State, Nigeria. BJOG: An International Journal of Obstetrics & Gynaecology. 2002;109(10): 1089-96.

89. Orji E, Babalola A. Correlates of female genital mutilation and its impact on safe motherhood. Journal of the Turkish German Gynecology Association. 2006;7(4): 319-24.

90. Paliwal P, Ali S, Bradshaw S, Hughes A, Jolly K. Management of type III female genital mutilation in Birmingham, UK: a retrospective audit. Midwifery. 2014;30(3): 282-8.

91. Penna C, Fallani MG, Fambrini M, Zipoli E, Marchionni M. Type III female genital mutilation: clinical implications and treatment by carbon dioxide laser surgery. American Journal of Obstetrics and Gynecology. 2002;187(6): 1550-4.

92. Pereda N, Arch M, Perez-Gonzalez A. A case study perspective on psychological outcomes after female genital mutilation. Journal of Obstetrics and Gynaecology. 2012;32(6): 560-5.

93. Plo K, Asse K, Seï D, Yenan J. Female genital mutilation in infants and young girls: report of sixty cases observed at the general hospital of abobo (abidjan, cote d’ivoire, west Africa). International Journal of Pediatrics. 2014: 1-5.

94. Raouf SA, Ball T, Hughes A, Holder R, Papaioannou S. Obstetric and neonatal outcomes for women with reversed and non‐reversed type III female genital mutilation. International Journal of Gynecology & Obstetrics. 2011;113(2): 141-3.

95. Rodriguez MI, Say L, Abdulcadir J, Hindin MJ. Clinical indications for cesarean delivery among women living with female genital mutilation. International Journal of Gynecology & Obstetrics. 2017;139(1): 21-7.

96. Rodriguez MI, Seuc A, Say L, Hindin MJ. Episiotomy and obstetric outcomes among women living with type 3 female genital mutilation: a secondary analysis. Reprod Health. 2016;13: 1-7.

97. Rouzi AA, Aljhadali EA, Amarin ZO, Abduljabbar HS. The use of intrapartum defibulation in women with female genital mutilation. BJOG: An International Journal of Obstetrics & Gynaecology. 2001;108(9): 949-51.

98. Rouzi AA, Al-Sibiani SA, Al-Mansouri NM, Al-Sinani NS, Al-Jahdali EA, Darhouse K. Defibulation during vaginal delivery for women with type III female genital mutilation. Obstetrics & Gynecology. 2012;120(1): 98-103.

99. Rouzi AA, Berg RC, Sahly N, Alkafy S, Alzaban F, Abduljabbar H. Effects of female genital mutilation/cutting on the sexual function of Sudanese women: a cross-sectional study. American Journal of Obstetrics and Gynecology. 2017;217(1): e1-e6.

100. Rouzi AA, Sahly N, Alhachim E, Abduljabbar H. Type I female genital mutilation: a cause of completely closed vagina. The Journal of Sexual Medicine. 2014;11(9): 2351-3.

101. Rouzi AA, Sindi O, Radhan B, Ba’aqeel H. Epidermal clitoral inclusion cyst after type I female genital mutilation. American Journal of Obstetrics and Gynecology. 2001;185(3): 569-71.

102. Saleh WF, Torky HA, Youssef MA, Ragab WS, Ahmed MAS, Eldaly A. Effect of female genital cutting performed by health care professionals on labor complications in Egyptian women: a prospective cohort study. Journal of Perinatal Medicine. 2018;46(4): 419-24.

103. Sayed GH, El-Aty MA, Fadel KA. The practice of female genital mutilation in Upper Egypt. International Journal of Gynecology & Obstetrics. 1996;55(3): 285-91.

104. Schiøtz HA, Bohlin T, Klingen TA, Aaberg T. Neuroma in clitoris after circumcision. Tidsskrift for Den Norske Laegeforening: Tidsskrift for Praktisk Medicin, Ny Raekke. 2012;132(6): 629-30.

105. Sharfi A, Elmegboul M, Abdella A. The continuing challenge of female genital mutilation in Sudan. African Journal of Urology. 2013;19(3): 136-40.

106. Slanger TE, Snow RC, Okonofua FE. The impact of female genital cutting on first delivery in southwest Nigeria. Studies in Family Planning. 2002;33(2): 173-84.

107. Théra T, Kouma A, Touré M, Coulibaly A, Sima M, Ongoiba I, Sagara A, et al. Obstetrical complications of genital mutilation in Malian rural environment. Journal de Gynecologie, Obstetrique et Biologie de la Reproduction. 2015;44(3): 276-279.

108. Torky HA, Elshenawy AA, Ahmad AM. Haematocolpos caused by a large vulvar dermoid cyst complicating type Ib female circumcision. Journal of Obstetrics and Gynaecology: the Journal of the Institute of Obstetrics and Gynaecology. 2018;38(4): 583.

109. Vangen S, Johansen REB, Sundby J, Træen B, Stray-Pedersen B. Qualitative study of perinatal care experiences among Somali women and local health care professionals in Norway. European Journal of Obstetrics & Gynecology and Reproductive Biology. 2004;112(1): 29-35.

110. Varol N, Dawson A, Turkmani S, Hall JJ, Nanayakkara S, Jenkins G, et al. Obstetric outcomes for women with female genital mutilation at an Australian hospital, 2006–2012: a descriptive study. BMC Pregnancy and Childbirth. 2016;16(1): 328.

111. WHO. Female genital mutilation and obstetric outcome: WHO collaborative prospective study in six African countries. Lancet (London, England). 2006;367(9525): 1835-41.

112. Wuest S, Raio L, Wyssmueller D, Mueller M, Stadlmayr W, Surbek D, et al. Effects of female genital mutilation on birth outcomes in Switzerland. BJOG: An International Journal of Obstetrics & Gynaecology. 2009;116(9): 1204-9.

113. Yassin K, Idris HA, Ali AA. Characteristics of female sexual dysfunctions and obstetric complications related to female genital mutilation in Omdurman maternity hospital, Sudan. Reproductive Health. 2018;15(1): 7.

114. Zayed AA, Ali AA. Abusing female children by circumcision is continued in Egypt. Journal of Forensic and Legal Medicine. 2012;19(4): 196-200.

115. Zoorob D, Kristinsdottir K, Klein T, Seo-Patel S. Symptomatic Clitoral Neuroma within an Epidermal Inclusion Cyst at the Site of Prior Female Genital Cutting. Case Reports in Obstetrics and Gynecology. 2019: 1-4.

116. Zurynski Y, Phu A, Sureshkumar P, Cherian S, Deverell M, Elliott EJ. Female genital mutilation in children presenting to Australian paediatricians. Archives of Disease in Childhood. 2017;102(6): 509-15.
